# Ionic liquid immobilized on modified magnetic FSM-16: an efficient and magnetically recoverable nanocatalyst†

**DOI:** 10.1039/d3ra04953a

**Published:** 2023-10-25

**Authors:** Azar Jahanbakhshi, Mahnaz Farahi

**Affiliations:** a Department of Chemistry, Yasouj University Yasouj 75918-74831 Iran farahimb@yu.ac.ir +98-7412242167e

## Abstract

In the present article, a nanocomposite was prepared by immobilizing ionic liquid on the magnetic mesoporous FSM-16 with a core–shell structure (Fe_3_O_4_@FSM-16-SO_3_/IL). Subsequently, the structural properties of the synthesized nanocatalyst were characterized and analyzed by various techniques such as XRD, FT-IR, TEM, FE-SEM, BET, VSM, TGA, and EDS. Fe_3_O_4_@FSM-16-SO_3_/IL was used as a recoverable and efficient nanocatalyst for the synthesis of polyhydroquinoline derivatives. The magnetic nanocatalyst showed remarkable stability and reusability and was reused six consecutive times without considerable loss of its activity.

## Abstract 2

In the present article, a nanocomposite was prepared by immobilizing ionic liquid on the magnetic mesoporous FSM-16 with a core–shell structure (Fe_3_O_4_@FSM-16-SO_3_/IL).

## Graphical abstract

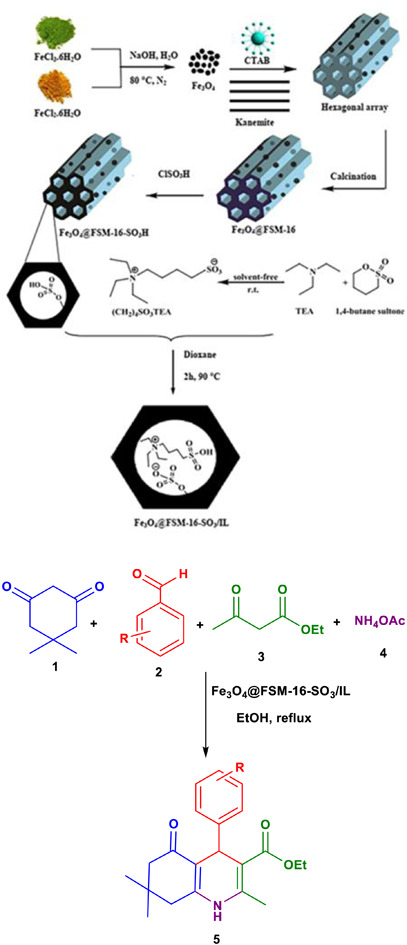

## 1. Introduction

In recent years, various types of mesoporous materials with high surface area, high pore volume, and large pore size have been synthesized using different surfactant templating procedures to expand their applications in the field of heterogeneous catalysts.^1,2^ Among the different mesoporous materials with structure and very regular pores, MCM-41 and FSM-16 have attracted much attention due to their many applications in various fields such as drug delivery, chromatography, adsorbents, electrodes, and also catalysts.^3–8^ FSM-16 structures have been prepared by intercalation of quarterly ammonium surfactant as a template into layered sodium silicate, kanemite, followed by calcination to remove the template, resulting in the formation of a hexagonal arrangement of channels with active surface silanol groups, high surface area, and uniform pore size.^9–11^ Some important studies reported on the FSM-16 based nanocatalysts are FSM-16@imine-thiophen/Pd,^12^ Fe_3_O_4_@FSM-16-SO_3_H,^13^ FSM-16-Met,^14^ FSM-16-SO_3_H,^15^ and FSM-16/AEPC-SO_3_H.^16^ Although porous nanomaterials have been widely used as supports for the preparation of heterogeneous catalysts, one of the major problems of them is the difficulty of separation from the reaction mixture by filtration and centrifugation. The key to overcoming these limitations is the use of magnetic nanoparticles (MNPs), which allow easy and faster separation of the porous nanomaterials from the reaction mixture by an external magnetic field.^17–19^ The combination of the properties of magnetic nanoparticles and mesoporous materials in a single material is particularly attractive from the point of view of catalysis because of possibility of combining the various functional groups with the advantages of the magnetic properties of magnetic nanoparticles. Therefore, magnetic mesoporous materials have attracted considerable attention as an attractive support for heterogeneous catalysts due to their superior properties such as environmentally benign nature, high stability, uniform porosity, easy separation, and recycling by applying a permanent magnet.^20–22^

New and innovative research in the field of green chemistry extensively discusses waste and damage reduction, innate atom economy, energy conservation, ease of use and the avoidance of perilous chemicals. Another important aspect of green chemistry is the use of ionic liquids (ILs) as new, green, and environmentally friendly solvents compared to toxic solvents for synthetic chemistry. In addition, ILs have attracted a wide application in the field of catalysis chemistry due to their unique properties such as high ionic conductivity, non-flammability, tunable acidity and basicity, high selectivity, high thermal stability, and reusability, and have been used in many organic reactions.^23–26^ The zwitterionic salts (ZIs) are organic salts that are composed of covalently bonded cations and anions. Zwitterions do not dissociate into ions, thus avoiding unwanted partitioning of ions can be avoided. The intramolecular covalent bonding of the cationic and anionic sites of zwitterions makes them stronger hydrogen bond donors or acceptors than their ionic liquid counterparts.^19,27,28^ Another interesting feature of ZIs is their ability to act as precursors for Brønsted acidic ionic liquids, which are widely used as solvents and catalysts in various organic reactions. However, the problems of recyclability and high viscosity have limited the use of ionic liquids use in catalytic reactions. A combination of interesting properties of the ionic liquid with those of the support material will develop novel performances when synergistic effects occur. Therefore, immobilization of ionic liquids on suitable solid supports such as porous supports or magnetic nanoparticles would be a short cut to achieve better catalytic efficiency and recycling performance than current ionic liquid catalysts.^29–33^ To date, many ionic liquids supported on magnetic mesoporous materials have been prepared and used in many chemical reactions. Some of the recently developed systems are Fe_3_O_4_@silica-MCM-41@DABCO,^34^ Fe_3_O_4_@MCM-41@IL/Pd,^35^ MCM-41@ILLaCl_4_,^36^ I@BFPMO-IL,^37^ Fe_3_O_4_@MCM-41@ZrCl_2_,^38^ Fe_3_O_4_@MCM-41-SO_3_H@[HMIm][HSO_4_],^39^ Fe_3_O_4_@MCM@IL-WO_4_^=^,^40^ SBA-15/NHSO_3_H,^41^ and Fe_3_O_4_@SiO_2_-HMTA-SO_3_H.^42^ The heterogenization of IL catalysts can also be achieved by the construction of poly(ionic liquid)s, and several catalysts of this type have been reported.^43–45^

Nitrogen-containing heterocycles often play an important role as the scaffolds for pharmacological compounds. Polyhydroquinoline derivatives, as an important class of N-heterocyclic compounds have become an important structural component in many pharmaceutical agents such as HIV protease inhibition, antitumor, neurotropic, antibacterial, MDR reversal, antimutagenic, antidiabetic, hepatoprotective, and vasodilator.^46–48^ Therefore, considering the medicinal and biological significance, the synthesis of this class of heterocyclic compounds has attracted considerable attention from synthetic chemists. Polyhydroquinoline derivatives are usually synthesized *via* the four-compound one-pot reaction of aldehyde, dimedone, ethyl acetoacetate, ammonium acetate, and the corresponding catalyst.^49–51^

In continuation of our studies program for the synthesis of novel heterogeneous nanocatalysts and also considering the importance of mesoporous materials in the catalysis synthesis process,^12,52–58^ herein, a recyclable magnetic catalyst based on FSM-16 (Fe_3_O_4_@FSM-16-SO_3_/IL) has been synthesized and characterized. Furthermore, Fe_3_O_4_@FSM-16-SO_3_/IL was used as an effective catalyst in the synthesis of polyhydroquinoline derivatives *via* the one-pot reaction of various aromatic aldehydes, dimedone, ethyl acetoacetate, and ammonium acetate.

## 2. Experimental

### 2.1. General

All solvents, chemicals, and reagents were purchased from Fluka, Merck, and Sigma-Aldrich companies. The purchased materials including FeCl_3_·6H_2_O, FeCl_2_·4H_2_O, sodium hydroxide (NaOH), cetyltrimethyl ammonium bromide (CTAB), tetraethyl *ortho* silicate (TEOS), cholorosulfunic acid (ClSO_3_H), triethyl amine (TEA), 1,4-butane sultone, dimedone, ethyl acetoacetate, ammonium acetate, benzaldehyde, 4-nitrobenzaldehyde, 4-chlorobenzaldehyde, 4-methylbenzaldehyde, 3-methoxybenzaldehyde, 3-bromobenzaldehyde, 2-chlorobenzaldehyde, 2,4-chlorobenzaldehyde, 2-hydroxy-3-methoxy benzaldehyde, ethanol, toluene, 1,4-dioxane, and dichloromethane, and were used without further purification. X-ray diffraction (XRD) analysis was investigated using a Rigaku Ultima IV diffractometer. Field emission-scanning electron microscopy (FE-SEM) images were recorded using FE-SEM TESCAN MIRA3. The energy-dispersive X-ray (EDX) spectrum was measured using a TESCAN VEGA model. Thermo gravimetric (TGA) analysis was performed using a PerkinElmer STA 6000 instrument. Transmission electron microscopy (TEM) images were examined using Philips EM208S instrument. The surface area was evaluated using the Brunauer–Emmett–Teller (BET) technique. The magnetic properties of the materials were monitored using a vibrating sample magnetometer (VSM) model MDK VSM. The FT-IR spectra in the range of 400–4000 cm^−1^ were obtained using FT-IR JASCO-Model 680 spectroscopy. The NMR spectroscopy was performed on a Bruker 400 MHz Ultrashield instrument at 400 MHz (^1^H-NMR) and 100 MHz (^13^C-NMR) in DMSO-*d*_6_ as solvent.

### 2.2. Synthesis of Fe_3_O_4_ nanoparticles

Initially, Fe_3_O_4_ NPs were prepared by adding FeCl_3_·6H_2_O (2.7 g, 10 mmol) and FeCl_2_·4H_2_O (1 g, 5 mmol) to deionized water (50 mL), followed by a dropwise addition of sodium hydroxide solution (5 mL, 10 M) with stirring at 80 °C under an N_2_ atmosphere for 1 h. The magnetic precipitate was then collected with a magnet and washed through deionized water and ethanol. The precipitate obtained was dried in an oven at 70 °C for 2 h.^54^

### 2.3. Synthesis of Fe_3_O_4_@FSM-16

Sodium hydroxide (3 g) was dissolved in deionized water (30 mL). Tetraethyl *ortho* silicate (TEOS) (16.6 mL) was added dropwise to the NaOH solution over 1 h and the resulting mixture was stirred for 24 h at 80 °C. The mixture was centrifuged and washed with deionized water, and dried at 80 °C for 12 h. The resulting product was then calcined in an oven at 650 °C for 5 h, and finally, the product of kanemite (δ-Na_2_Si_2_O_5_) was synthesized. Kanemite (5 g) was added to deionized water (50 mL) and stirred at 30 °C for 3 h. The resulting suspension was then filtered to obtain the wet kanemite dough. Next, Fe_3_O_4_ nanoparticles (0.106 g, 0.457 mmol) were dispersed in deionized water (50 mL) by ultrasonic waves and cetyltrimethylammonium bromide (CTAB) was added to this solution by slowly raising the temperature to 70 °C. Then, the kanemite paste was added and then stirred at 70 °C for 3 h. At this stage, the pH value of the suspension was 11.5–12.5. After 3 h, the pH of the medium was adjusted to 8.5 with HCl (2 M), and the mixture was stirred at 70 °C for another 3 h. After cooling the mixture, the resulting solid was separated by centrifugation and washed with deionized water. The magnetite mesoporous silicate (Fe_3_O_4_@FSM-16) was dried in an oven at 80 °C for 2 h and then calcined in an oven at 650 °C for 5 h to burn the surfactant and synthesis of the final magnetite mesoporous silicate, Fe_3_O_4_@FSM-16.^13^

### 2.4. Synthesis of Fe_3_O_4_@FSM-16-SO_3_H

Fe_3_O_4_@FSM-16 (0.5 g) was then sonicated for 15 min in dry CH_2_Cl_2_ (5 mL) in a 10 mL round bottom flask. Then cholorosulfunic acid (ClSO_3_H) (0.15 mL) was added dropwise to the reaction mixture for 15 min at room temperature. The reaction mixture was stirred for 2 h. Finally, the brown solid was separated with an external magnet and was dried in an oven at 100 °C for 2 h to obtain Fe_3_O_4_@FSM-16-SO_3_H.^13^

### 2.5. Preparation of zwitterionic salts [(CH_2_)_4_SO_3_TEA]

The zwitterionic solid was synthesized by a one-step reaction between triethylamine (1 mmol) and 1,4-butane sultone (1 mmol) under solvent-free conditions and stirring at room temperature for 24 h. The resulting solid salt was washed several times with diethyl ether and dried in a vacuum at 60 °C.^59^

### 2.6. Electrostatic immobilization of ionic liquid on Fe_3_O_4_@FSM-16-SO_3_H [Fe_3_O_4_@FSM-16-SO_3_/IL]

Ultimately, Fe_3_O_4_@FSM-16-SO_3_H groups (1 g) in toluene (20 mL) were dispersed by sonication for 15 min and [(CH_2_)_4_SO_3_TEA] (0.6 g) in 1,4-dioxane (15 mL) was added to the mixture at room temperature. The temperature of the reaction was then slowly increased to 85 °C and stirred for 3 h. The reaction solvent was removed under a vacuum at 85 °C. The Fe_3_O_4_@FSM-16-SO_3_/IL catalyst obtained was washed with ethanol and dried under vacuum at 65 °C.

### 2.7. General procedure for the synthesis of polyhydroquinoline derivatives using Fe_3_O_4_@FSM-16-SO_3_/IL nanocatalyst

A mixture of aldehyde (1 mmol), dimedone (1 mmol), ethyl acetoacetate (1 mmol), ammonium acetate (1.2 mmol), and Fe_3_O_4_@FSM-16-SO_3_/IL nanocatalyst (0.004 g) in ethanol (5 mL) was stirred under reflux conditions. The progress of the reaction was monitored by TLC (*n*-hexane/ethyl acetate, 2 : 1).

At the end of the reaction, the Fe_3_O_4_@FSM-16-SO_3_/IL nanocatalyst was separated by an external magnet. Finally, the pure products were purified by recrystallization from MeOH.

### 2.8. Recovery

Fe_3_O_4_@FSM-16-SO_3_/IL (0.004 g) was added to the mixture of aldehyde (1 mmol), dimedone (1 mmol), ethyl acetoacetate (1 mmol) and ammonium acetate (1.2 mmol) in (5 mL) ethanol and stirred under reflux conditions. After completion of the reaction, hot methanol (10 mL) was added to the reaction mixture; the catalyst was separated by an external magnet, washed three times with MeOH, and dried. The isolated catalyst was used directly for the subsequent runs under similar conditions.

## 3. Results and discussion

### 3.1. Synthesis and characterization of the catalyst

**Scheme 1:**
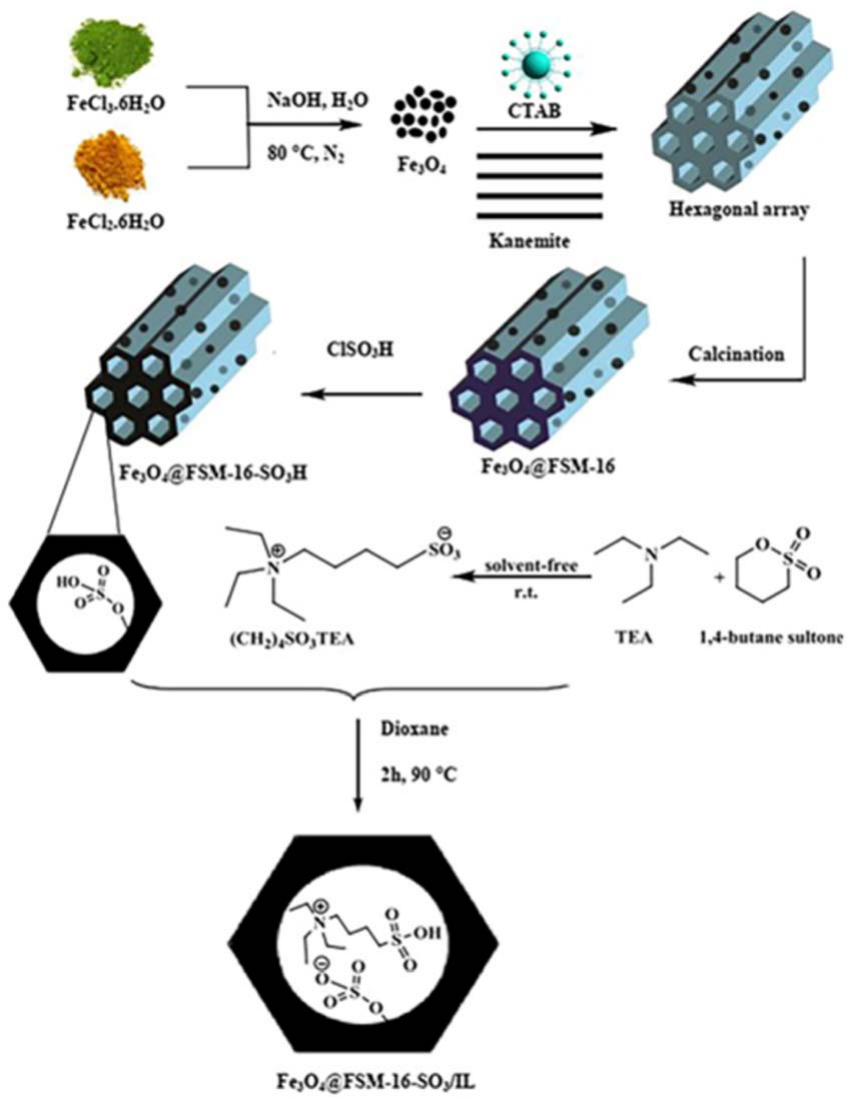
Functionalization of FSM-16 and electrostatic immobilization of IL.

In this work, for the preparation of Fe_3_O_4_@FSM-16-SO_3_/IL, the acidic ionic liquid immobilization on Fe_3_O_4_@FSM-16 nanocomposite was performed in several steps as shown in Scheme 1. Firstly, Fe_3_O_4_ nanoparticles were prepared and then coated with CTAB and kanemite (δ-Na_2_Si_2_O_5_). The resulting product was calcined to produce Fe_3_O_4_@FSM-16. Subsequently, Fe_3_O_4_@FSM-16-SO_3_H was prepared by the reaction of between Fe_3_O_4_@FSM-16 with chlorosulfunic acid. Then, the reaction of triethylamine with 1,4-butane sultone produced the ionic liquid (IL). Finally, the Fe_3_O_4_@FSM-16-SO_3_/IL nanocatalyst was prepared by the electrostatic stabilization the IL on Fe_3_O_4_@FSM-16-SO_3_H. The synthesized nanocatalyst was characterized by XRD, FT-IR, TGA, FE-SEM, EDS, TEM, BET, and VSM analyses.

**Fig. 1:**
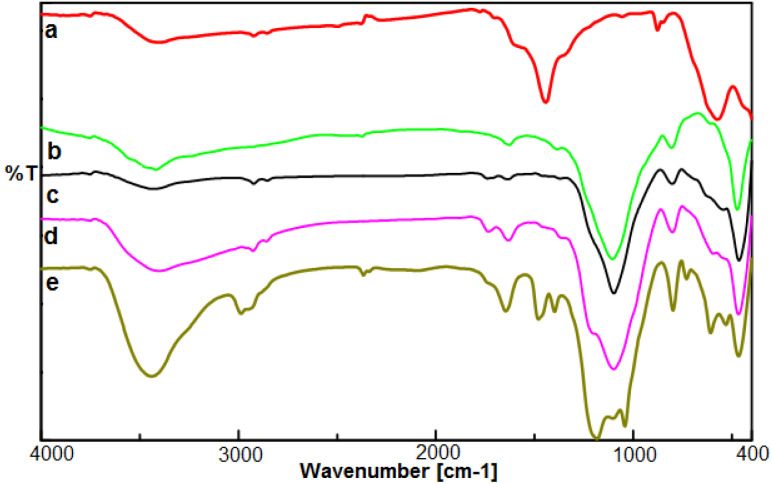
The FT-IR spectrum of (a) Fe_3_O_4_, (b) FSM-16, (c) Fe_3_O_4_@FSM-16, (d) Fe_3_O_4_@FSM-16-SO_3_H, and (e) Fe_3_O_4_@FSM-16-SO_3_/IL.

The FT-IR spectra of Fe_3_O_4_, FSM-16, Fe_3_O_4_@FSM-16, Fe_3_O_4_@FSM-16-SO_3_H, and Fe_3_O_4_@FSM-16-SO_3_/IL were compared to analyze the progress of the catalyst synthesis (Fig. 1). In Fig. 1a, the absorption bands revealed at 574 and 3400 cm^−1^ are attributed to Fe–O and O–H bonds, respectively.^54,56^ Also, in Fig. 1b, the peaks at 474, 806, and 1106 cm^−1^ correspond to asymmetric and symmetric stretching vibrations of the Si–O–Si group.^12^ In Fig. 1c, the peaks of the Fe–O bond at 588 cm^−1^ overlap with the S–O bond at about 600 cm^−1^. The increase in the intensity of the peak at 1106 cm^−1^ is related to the O=S=O band, indicating the –SO_3_H group in the Fe_3_O_4_@FSM-16-SO_3_H (Fig. 1d).^39,41^ The stretching band at about 2950 cm^−1^ corresponds to the CH_2_ groups, and the appearing bands at 1400–1600 cm^−1^ are related to the stretching vibrations of C–C and C–N (Fig. 1e).^4,59^

**Fig. 2:**
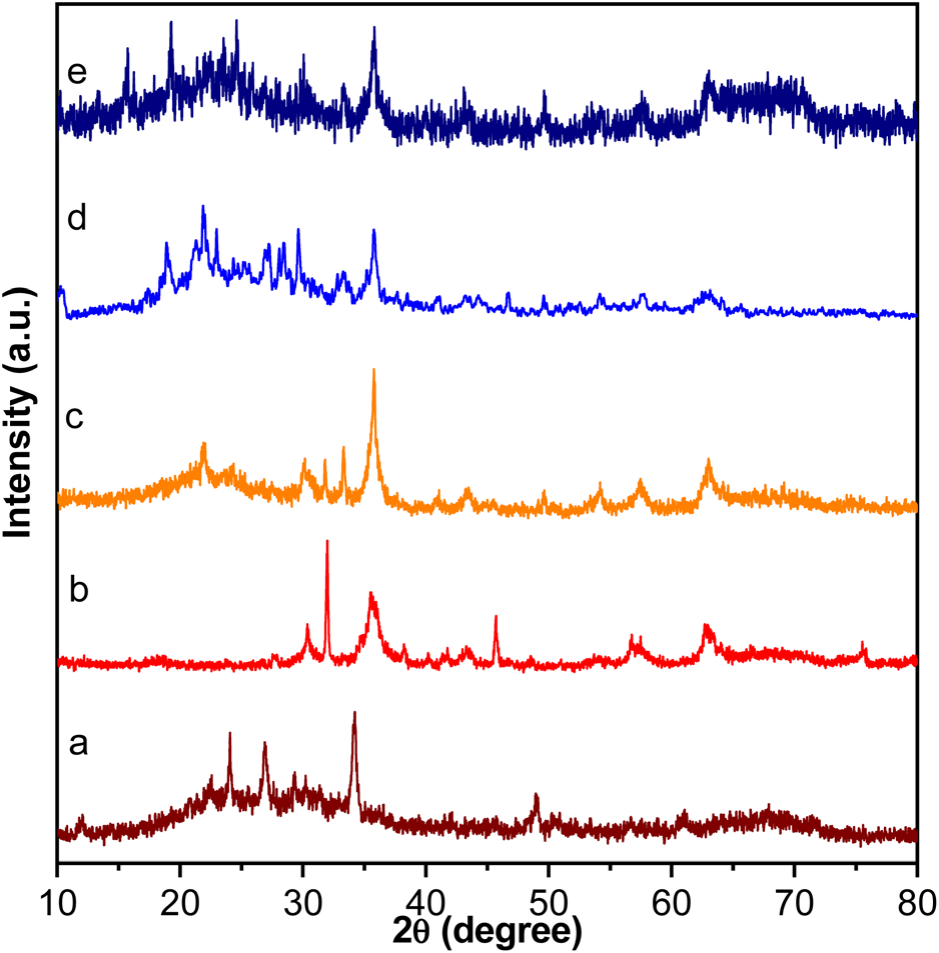
XRD patterns of (a) FSM-16, (b) Fe_3_O_4_, (c) Fe_3_O_4_@FSM-16, (d) Fe_3_O_4_@FSM-16-SO_3_H, and (e) Fe_3_O_4_@FSM-16-SO_3_/IL.

The XRD patterns of the FSM-16, Fe_3_O_4_, Fe_3_O_4_@FSM-16, Fe_3_O_4_@FSM-16-SO_3_H, and Fe_3_O_4_@FSM-16-SO_3_/IL in the range of 2*θ* = 10–80° are shown in Fig. 2. In Fig. 2a, the four characteristic peaks at 2*θ* = 23.4°, 37.1°, 48.5° and 61.5° related to the (100), (110), (200), and (210) planes of FSM-16 with regular hexagonal structures.^8,11,12^ In the XRD pattern of Fe_3_O_4_ nanoparticles, six peaks indicated at 2*θ* = 30.26°, 35.7°, 43.5°, 53.59°, 57.5°, and 63.26° related to the (2 2 0), (3 1 1), (4 0 0), (4 2 2), (5 1 1), and (4 4 0) which confirm the spinel structure of Fe_3_O_4_ (Fig. 2b).^54,56,57^ As shown in Fig. 2c, the XRD pattern of Fe_3_O_4_@FSM-16 is in good agreement with the XRD pattern of previous reports of Fe_3_O_4_ coated FSM-16 structures (Fe_3_O_4_@FSM-16).^13^ Furthermore, as shown in Fig. 2d, the peak intensities are slightly reduced, which is due to the stabilization of –SO_3_H acidic groups on the surface Fe_3_O_4_@FSM-16. According to Fig. 2e, the XRD pattern of the Fe_3_O_4_@FSM-16-SO_3_/IL nanocatalyst has been changed due to the immobilization of IL on Fe_3_O_4_@FSM-16-SO_3_H. Also, the comparison of Fig. 2e with Fig. 2b and c confirms the immobilization of Fe_3_O_4_, –SO_3_H, and IL on FSM-16, and shows that the Fe_3_O_4_@FSM-16-SO_3_/IL catalyst was successfully synthesized.

**Fig. 3:**
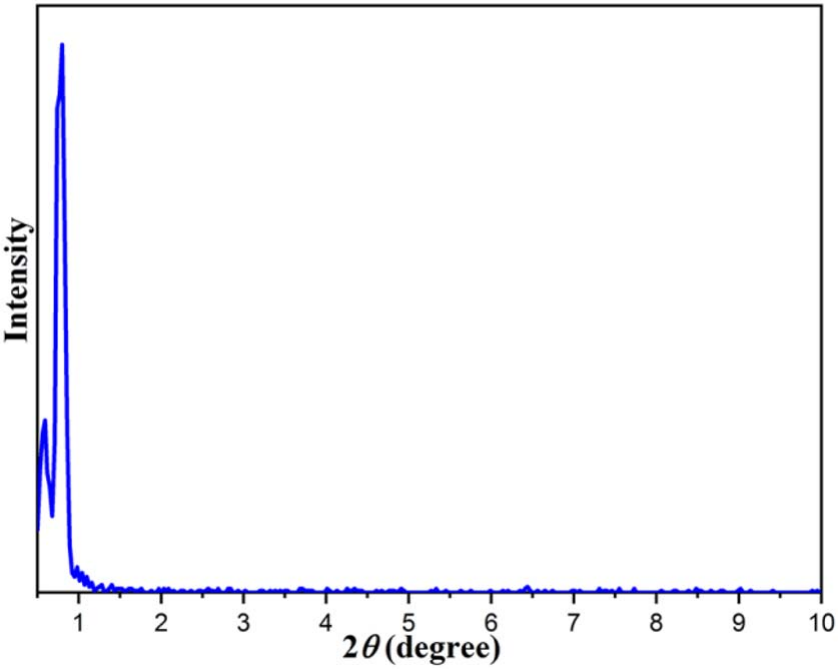
XRD low angle XRD patterns of Fe_3_O_4_@FSM-16-SO_3_/IL.

In addition, the low angle X-ray diffraction (LXRD) analysis of the Fe_3_O_4_@FSM-16-SO_3_/IL catalyst in Fig. 3 shows a high intensity peak at 2*θ* = 0.91° corresponding to (1 0 0), which is characteristic of hexagonally ordered mesoporous materials.

**Fig. 4:**
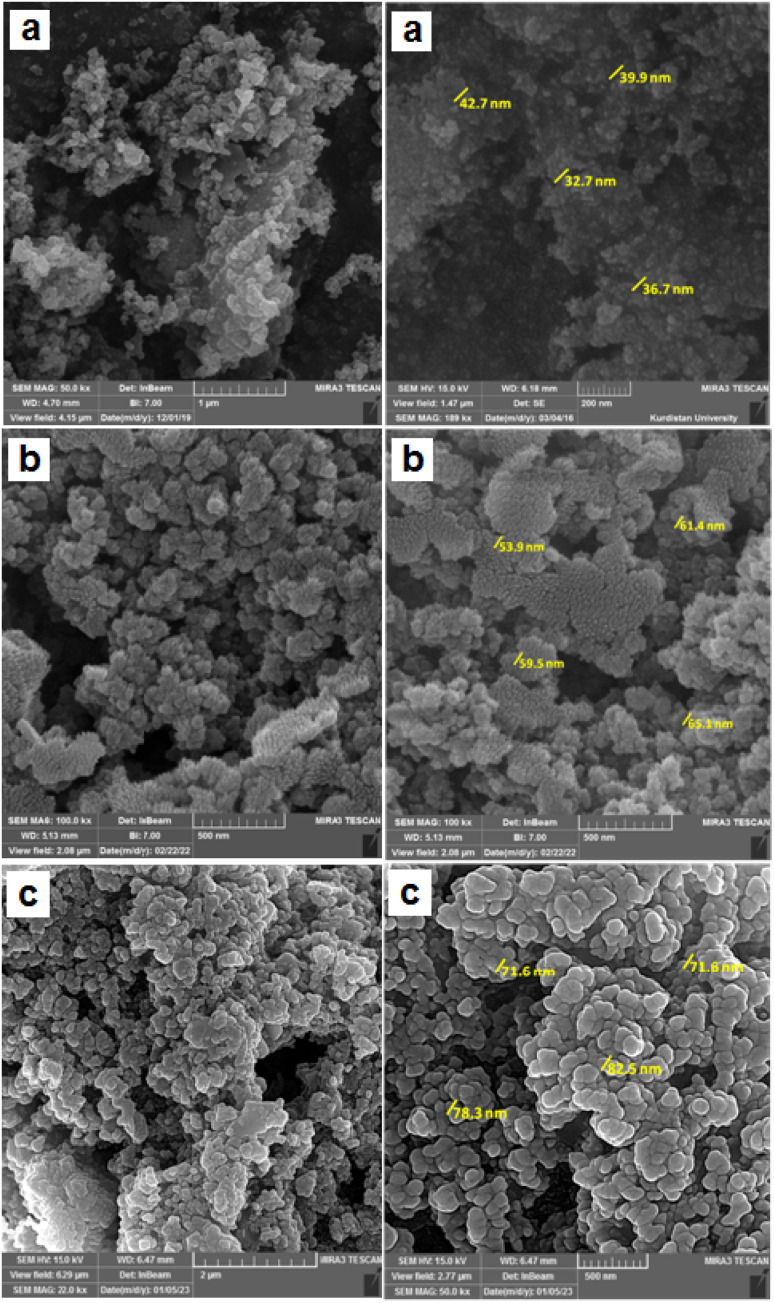
The FE-SEM images of (a) Fe_3_O_4_, (b) FSM-16 (image (b) has been reused from a previous publication, https://doi.org/10.1039/D2RA06271B),^12^ and (c) Fe_3_O_4_@FSM-16-SO_3_/IL.

The surface morphology, particle size and structural features of Fe_3_O_4_, FSM-16,^12^ and Fe_3_O_4_@FSM-16-SO_3_/IL nanocatalyst were identified by using field effect scanning electron microscopy (FE-SEM) (Fig. 4). The FE-SEM images show that the synthesized nanocatalyst has an almost regular spherical morphology and uniformity and is less than 100 nm in particle size. Furthermore, it is obvious from Fig. 4 that the average particle size of Fe_3_O_4_ and FSM-16 significantly changed after stabilization with SO_3_H and IL, which indicates the successful synthesis of Fe_3_O_4_@FSM-16-SO_3_/IL.

**Fig. 5:**
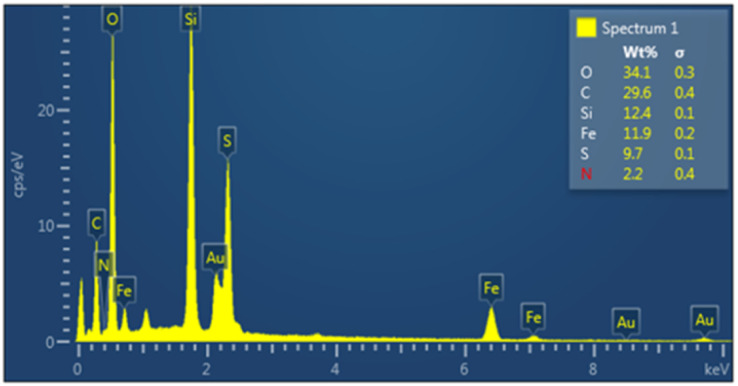
The EDX spectra of Fe_3_O_4_@FSM-16-SO_3_/IL.

As shown in Fig. 5, energy-dispersive X-ray (EDX) analysis was used to determine the elements of nanoporous Fe_3_O_4_@FSM-16-SO_3_/IL. The results confirm the presence of C, O, N, S, Si, and Fe in the synthesized nanocatalyst, and it could be inferred that the target catalyst was successfully synthesized.

**Fig. 6:**
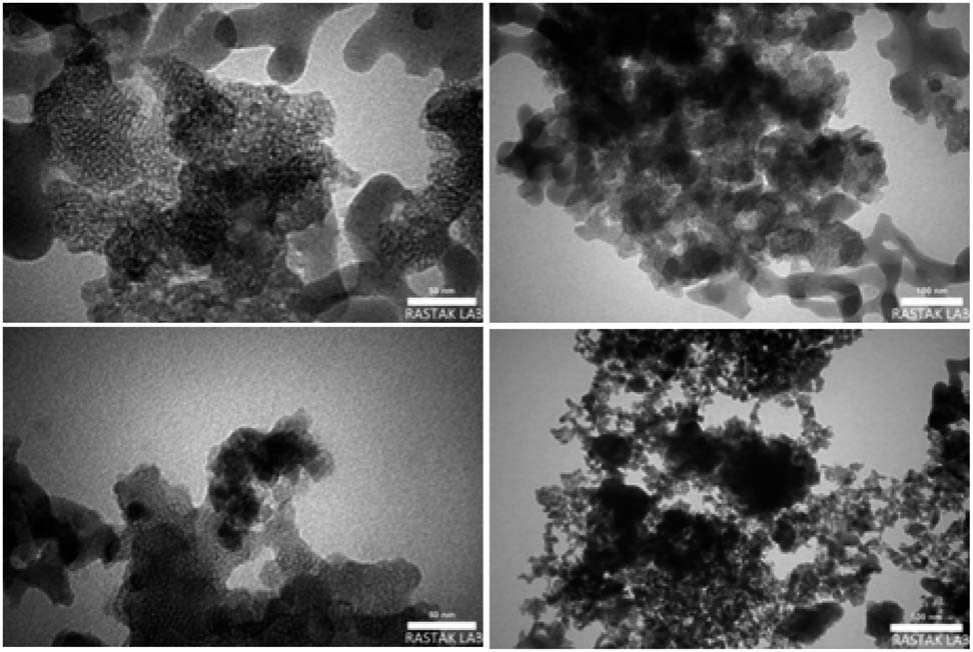
TEM images of Fe_3_O_4_@FSM-16-SO_3_/IL.

The TEM images of Fe_3_O_4_@FSM-16-SO_3_/IL catalyst are shown in Fig. 6. As seen in the images, the black cores of Fe_3_O_4_ NPs are surrounded by a grey shell of FSM-16-SO_3_/IL.

**Fig. 7:**
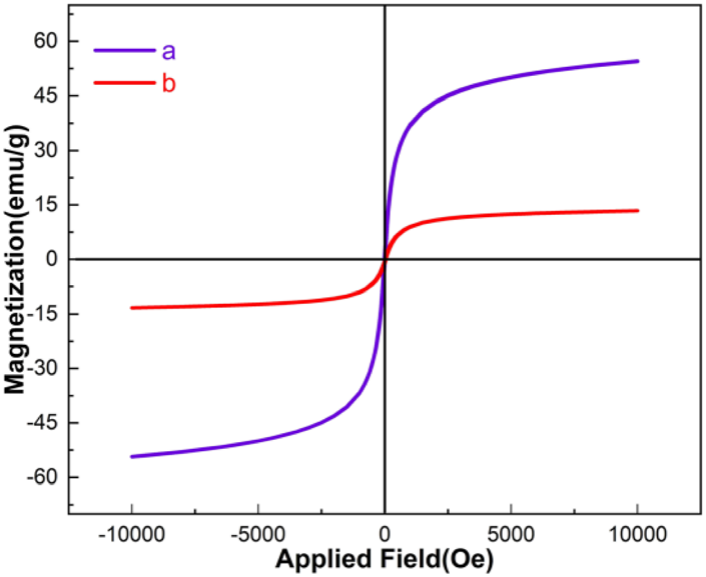
VSM curves of the (a) Fe_3_O_4_ and (b) Fe_3_O_4_@FSM-16-SO_3_/IL.

The magnetic properties of the Fe_3_O_4_ and Fe_3_O_4_@FSM-16-SO_3_/IL were investigated using the vibrating sample magnetometer (VSM) technique at room temperature (Fig. 7). Based on the VSM curves, the magnetization was measured to be Fe_3_O_4_ 53.03 emu g^−1^, and for Fe_3_O_4_@FSM-16-SO_3_/IL 15.5 emu per g nanocatalyst. It is essential to notice that the decrease in the magnetization value Fe_3_O_4_@FSM-16-SO_3_/IL nanocatalyst compared to Fe_3_O_4_ may be due to the immobilization of the shell of FSM-16-SO_3_H and then IL around the magnetic Fe_3_O_4_ cores. The results showed that even with the reduction of the magnetization; the catalyst can be successfully separated from the reaction mixture by an external magnet.

**Fig. 8:**
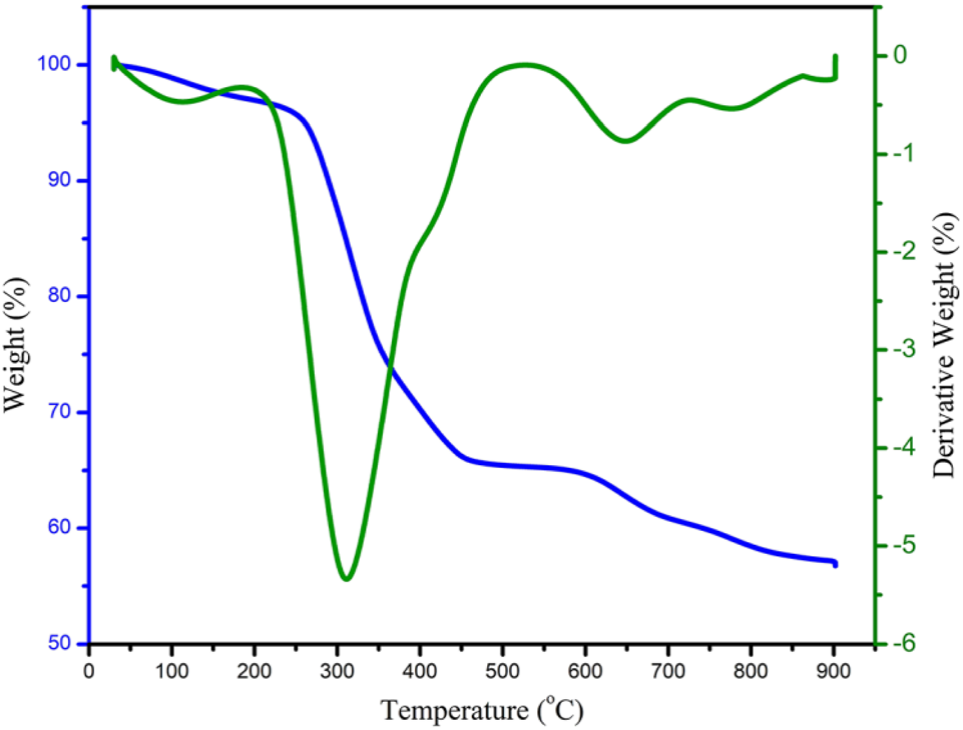
TGA analysis of the Fe_3_O_4_@FSM-16-SO_3_/IL.

To investigate the thermal stability of the Fe_3_O_4_@FSM-16-SO_3_/IL nanocatalyst, the thermo gravimetric analysis (TGA) was performed in the range of 25–900 °C (Fig. 8). Based on the TGA spectrum, the first weight loss of about 4.6% at a temperature below 220 °C was related to the removal of water, physically and chemically absorbed solvents, and surface hydroxyl groups on the Fe_3_O_4_@FSM-16-SO_3_/IL surface. The second and largest weight loss in the range of 220–480 °C is about 28%, related to the thermal decomposition of the sulfuric acid group, amine group, and organic groups on the surface of the Fe_3_O_4_@FSM-16 nanocomposite. The third and last weight loss of about 8% between 480 and 900 °C is related to the thermal complete decomposition of Fe_3_O_4_@FSM-16 and the ionic liquid (IL) stabilized on its surface, confirming the thermal stability of the prepared Fe_3_O_4_@FSM-16-SO_3_/IL nanocatalyst.

**Fig. 9:**
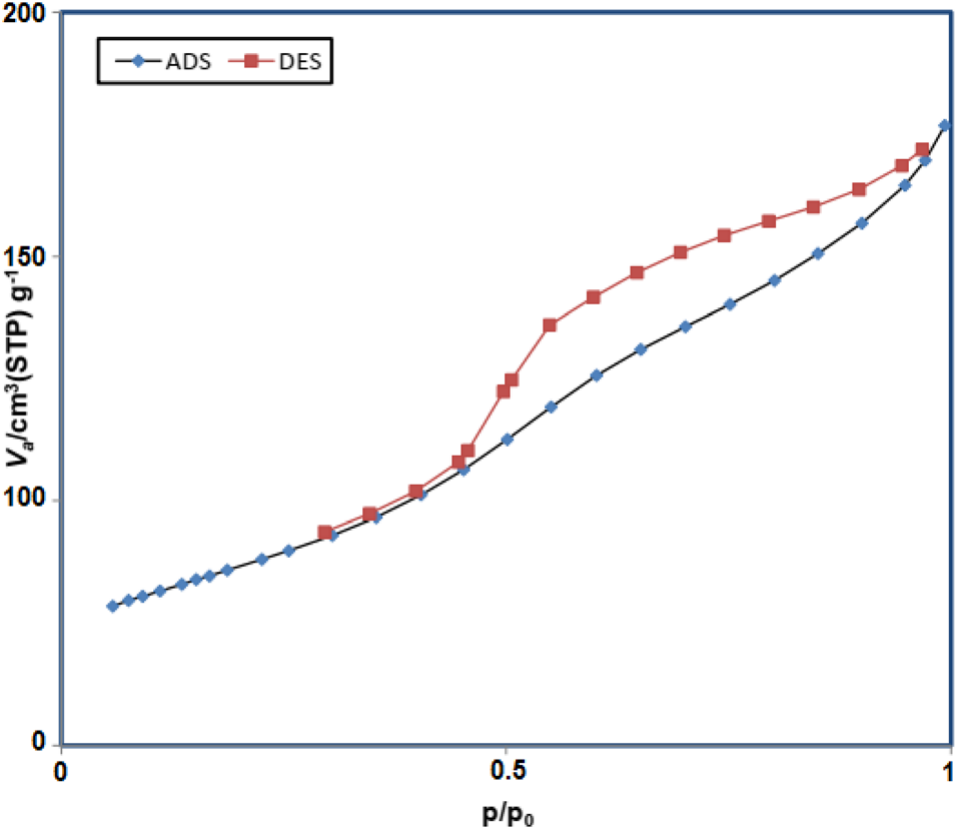
N_2_ adsorption–desorption isotherms of Fe_3_O_4_@FSM-16-SO_3_/IL.

In addition, based on the physicochemical and structural parameters of Fe_3_O_4_@FSM-16-SO_3_/IL nanocatalyst, the nitrogen adsorption–desorption isotherms were measured (Fig. 9). The type of N_2_ adsorption–desorption isotherm of prepared nanocatalyst, according to the IUPAC classification, is a type III isotherm, which indicating of the typical mesoporous structure. Furthermore, according to the Brunauer–Emmett–Teller (BET) analysis, the surface area, total pore volume, and mean pore diameter of the Fe_3_O_4_@FSM-16-SO_3_/IL catalyst 180 m^2^ g^−1^, 0.02 cm^3^ g^−1^, and 5.83 nm were obtained respectively.

### 3.2. Synthesis of polyhydroquinoline derivatives

**Scheme 2:**
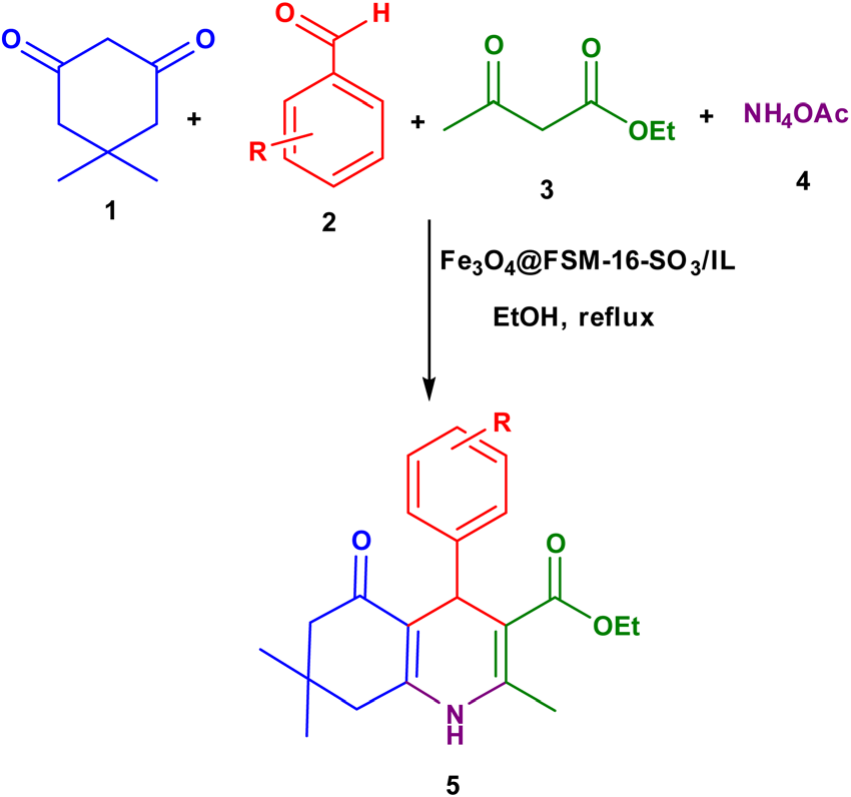
Synthesis of polyhydroquinoline derivatives 5 using Fe_3_O_4_@FSM-16-SO_3_/IL nanocatalyst.

After the successful characterization of the Fe_3_O_4_@FSM-16-SO_3_/IL nanocatalyst, its catalytic application was investigated in the synthesis of polyhydroquinoline derivatives with the multi-component reaction of aromatic aldehyde, dimedone, ethyl acetoacetate, and ammonium acetate (Scheme 2).

Initially, to obtain the best reaction conditions, a one-pot reaction between benzaldehyde, dimedone, ethyl acetoacetate, and ammonium acetate as a model reaction was investigated. For this purpose, the effect of catalyst loading, temperature, and solvent was studied. Primarily, the catalytic efficiency of the Fe_3_O_4_@FSM-16-SO_3_/IL nanocatalyst was investigated. The catalyst loading study showed that the use of 0.004 g of Fe_3_O_4_@FSM-16-SO_3_/IL gave the highest conversion. Then, to obtain the optimum solvent, the model reaction was carried out in different polar and non-polar solvents including toluene, CH_3_CN, MeOH, EtOH, H_2_O, DMSO, and also under solvent-free conditions. It was found that the best result was obtained in refluxed ethanol. The reaction temperature was also affected and the best result was observed at reflux conditions in ethanol. Finally, according to the mentioned results, the use of 0.004 g of Fe_3_O_4_@FSM-16-SO_3_/IL, and EtOH solvent at reflux conditions was selected as the optimum conditions (Table 1). Subsequently, different aryl aldehydes containing electron-donating and electron-accepting groups were used to prepare polyhydroquinoline derivatives. As shown in Table 2, the products were successfully obtained in good to excellent yields.

**Table 1:** Optimization of the reaction conditions for the synthesis of 5a^a^

Entry	Catalyst loading (g)	Solvent	Temp. (°C)	Yield^b^ (%)
1	0.002	—	80	55
2	0.004	—	80	94
3	0.005	—	80	94
4	0.007	—	80	94
5	0.004	EtOH	Reflux	96
6	0.004	H_2_O	80	65
7	0.004	CH_3_CN	80	55
8	0.004	DMSO	80	60
9	0.004	Toluene	80	45
10	0.004	EtOH	25	40
11	0.004	EtOH	50	60

a Reaction conditions: benzaldehyde (1 mmol), dimedone (1 mmol), ethyl acetoacetate (1 mmol), ammonium acetate (1.2 mmol). Time: 30 min.

b Isolated yields.

**Table 2:** Synthesis of derivatives 5 using Fe_3_O_4_@FSM-16-SO_3_/IL nanocatalyst^a^

Entry	Aldehyde	Product 5	M. p. (°C)	Yield^b^ (%)
5a	C_6_H_5_CHO	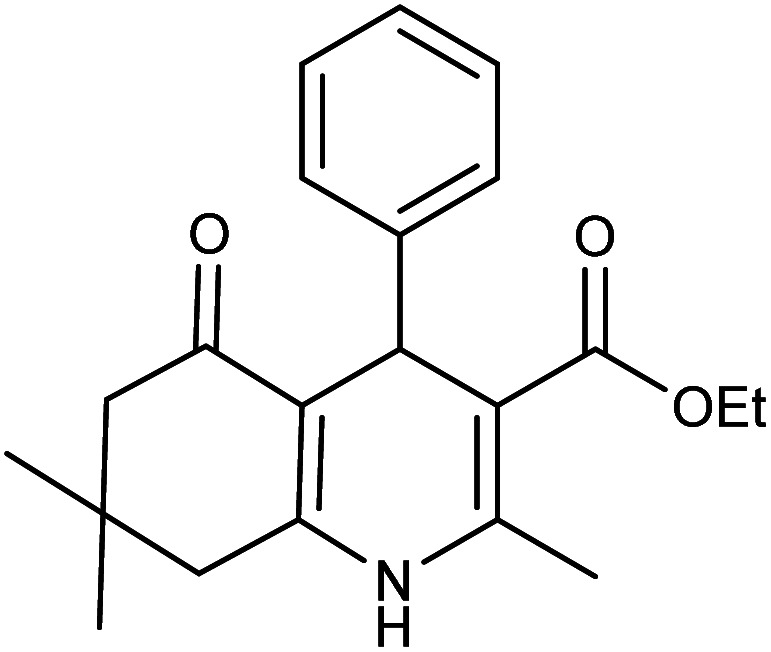	204–206 (ref. 65)	98
5b	4-OMe-C_6_H_4_CHO	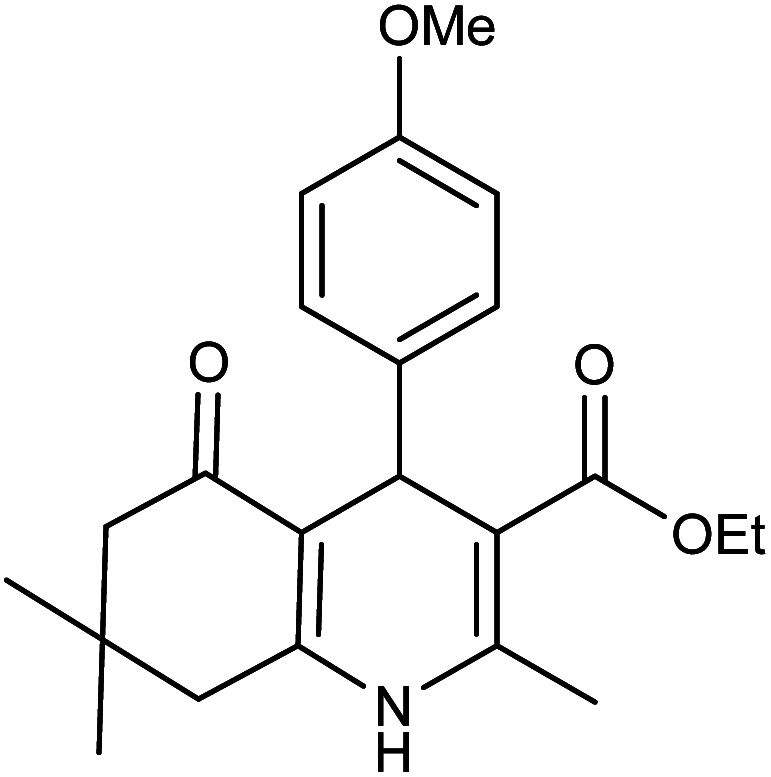	252–254 (ref. 65)	92
5c	4-NO_2_-C_6_H_4_CHO	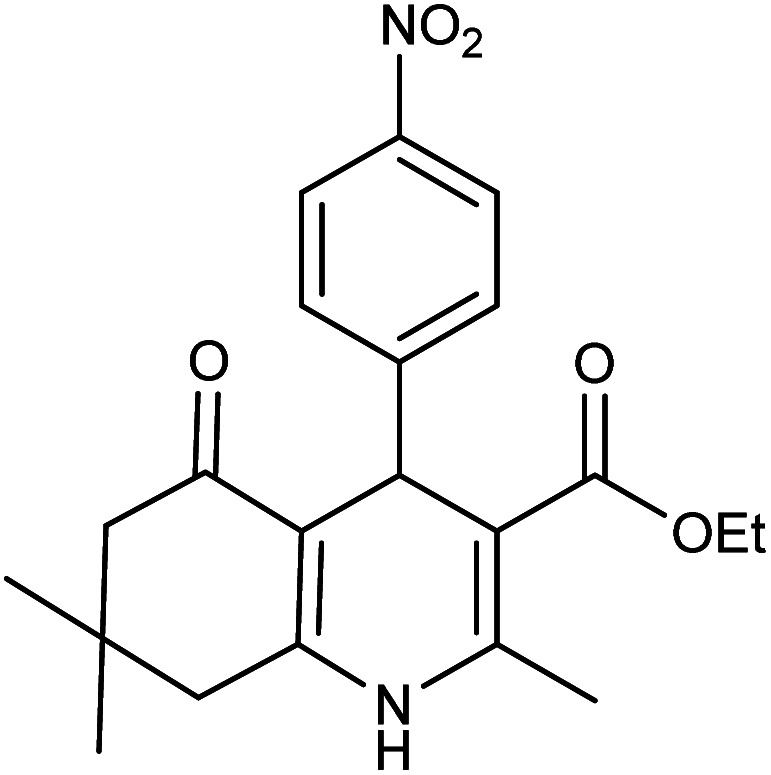	236–238 (ref. 65)	93
5d	4-Cl-C_6_H_4_CHO	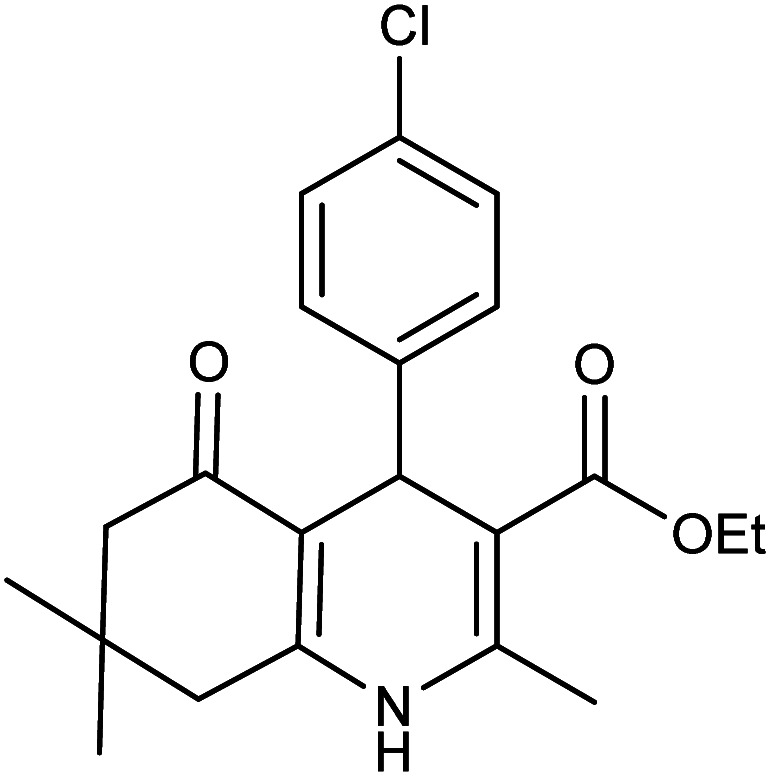	243–244 (ref. 60)	92
5e	2-Cl-C_6_H_4_CHO	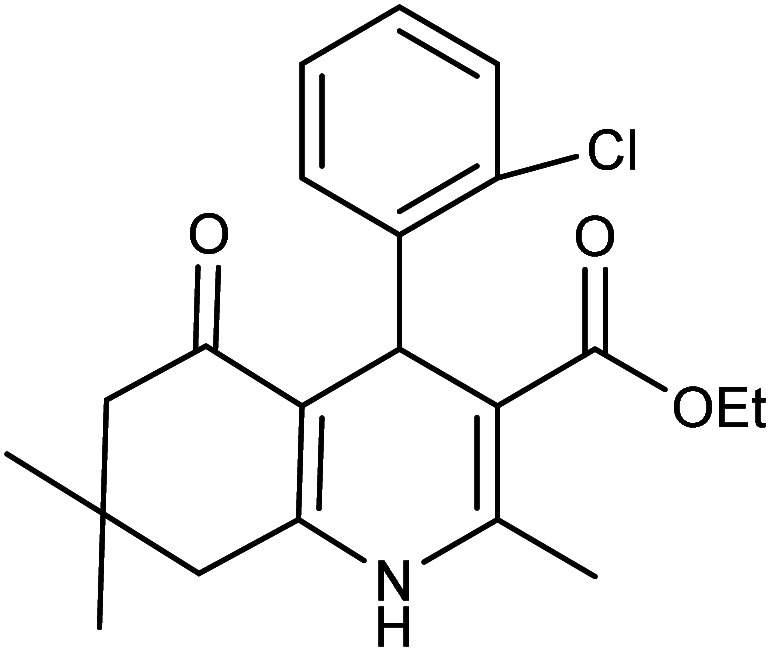	208–210 (ref. 60)	95
5f	2,4-Cl_2_-C_6_H_3_CHO	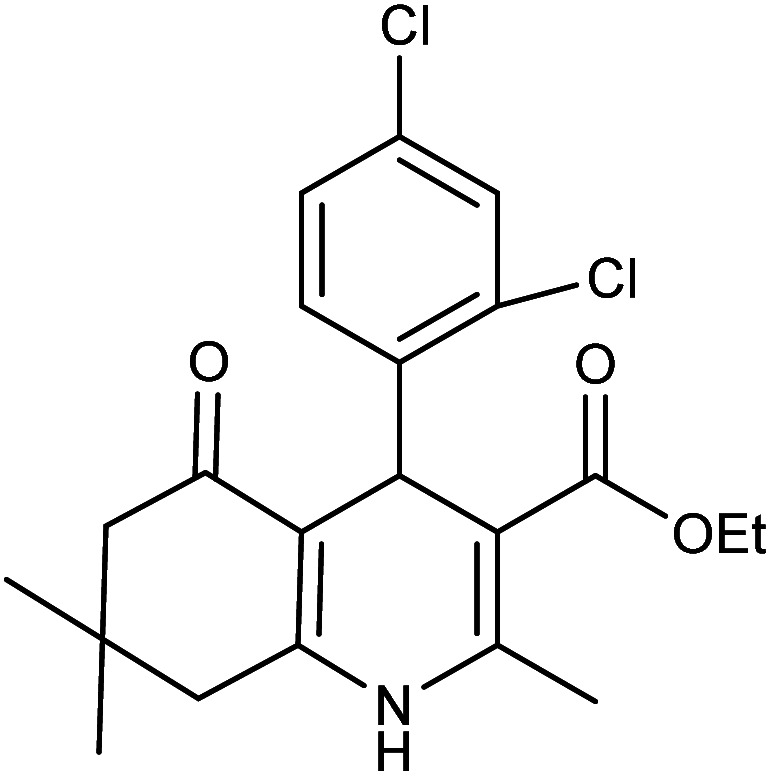	244–245 (ref. 62)	96
5g	3-Br-C_6_H_4_CHO	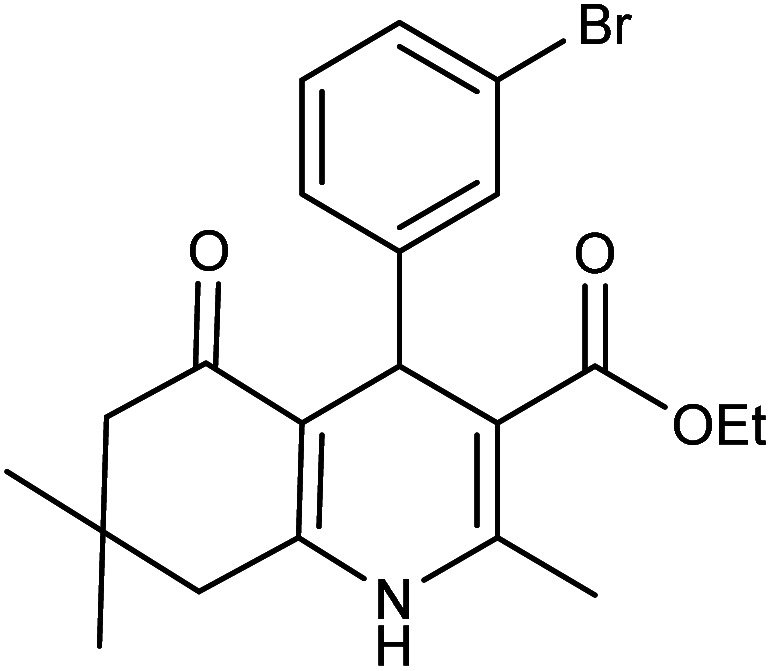	234–236 (ref. 62)	92
5h	4-Me-C_6_H_4_CHO	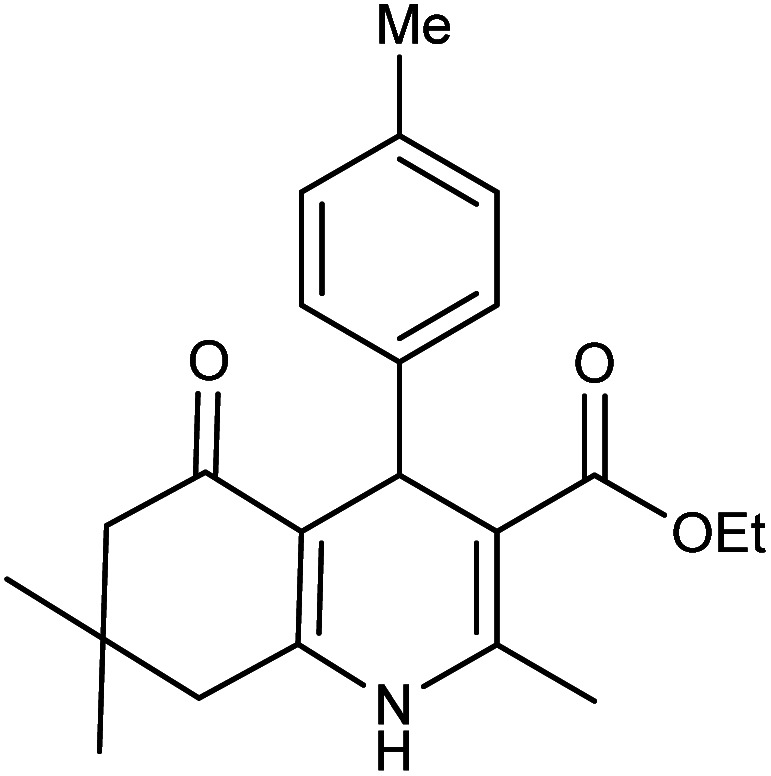	270–272 (ref. 62)	93
5i	2-OH-3-OMe-C_6_H_3_CHO	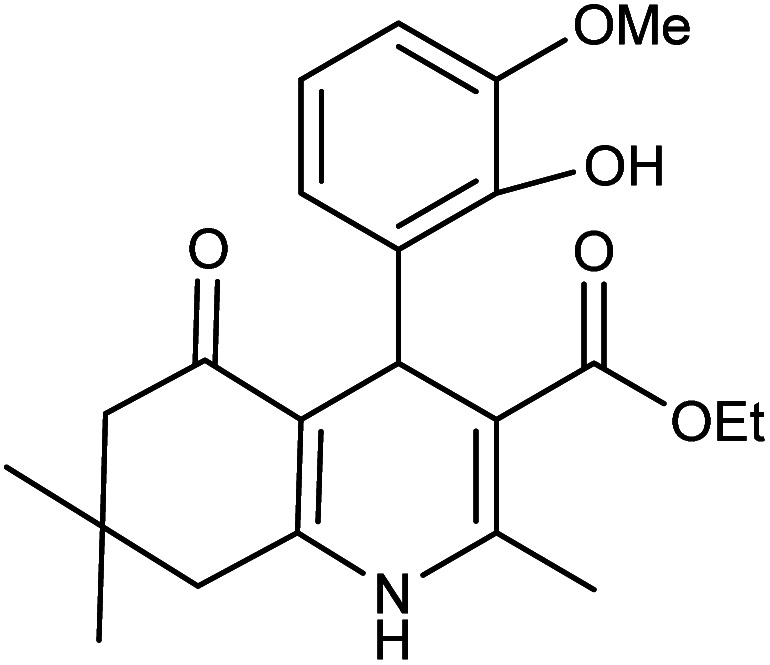	210–212 (ref. 63)	92

a Reaction conditions: aromatic aldehyde (1 mmol), dimedone (1 mmol), ethyl acetoacetate (1 mmol), ammonium acetate (1.2 mmol), Fe_3_O_4_@FSM-16-SO_3_/IL (0.004 g), EtOH (5 mL), reflux conditions. Time: 5–20 min.

b Isolated yields.

**Scheme 3:**
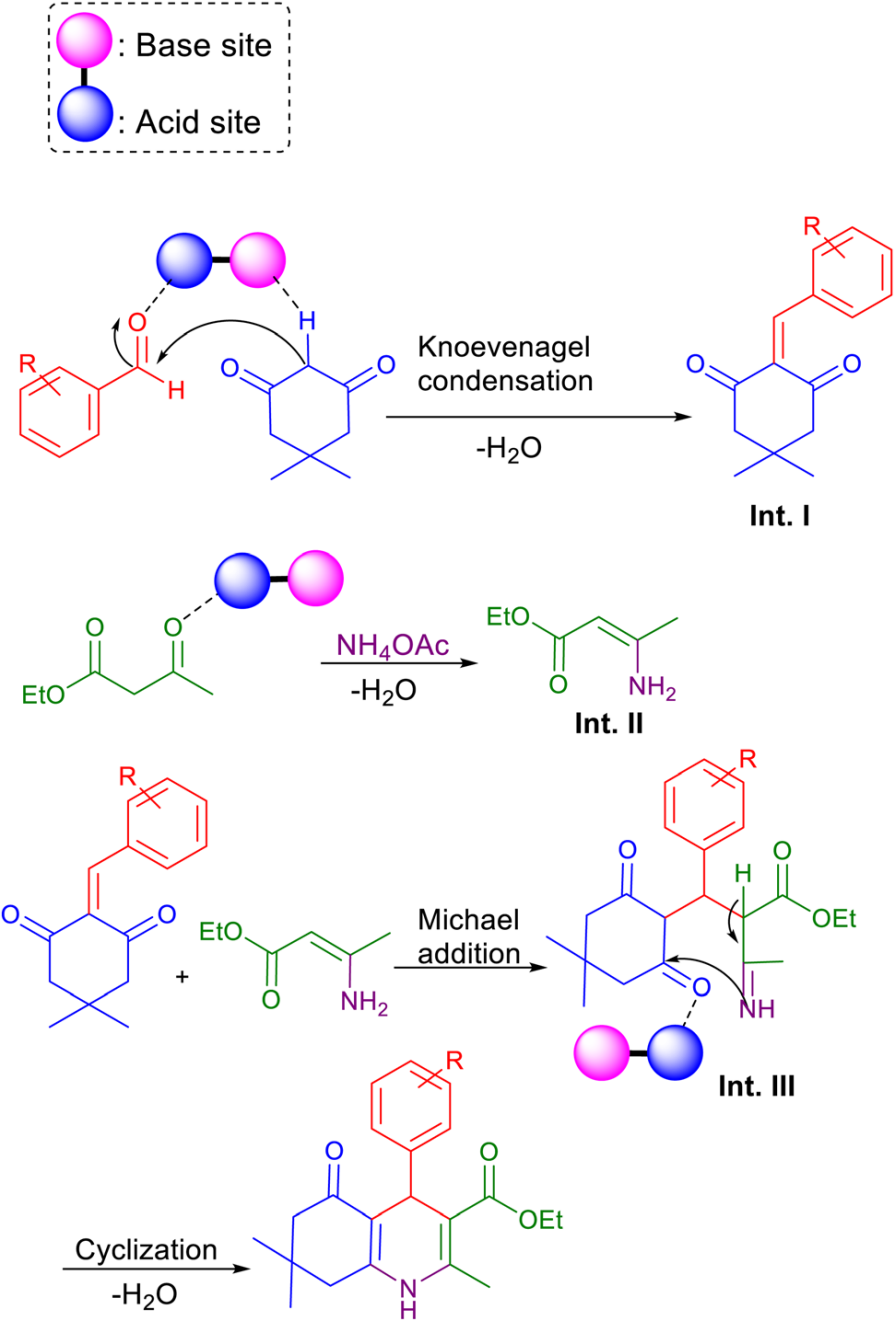
The suggested mechanism for the synthesis of polyhydroquinoline derivatives 5 using Fe_3_O_4_@FSM-16-SO_3_/IL.

As shown in Scheme 3, a mechanism for the synthesis of polyhydroquinoline derivatives (5a–i) *via* the Hantzsch reaction in the presence of Fe_3_O_4_@FSM-16-SO_3_/IL nanocatalyst is proposed. Firstly, dimedone and aldehyde were activated by the base site and the acidic sites of the nanocatalyst, respectively. Then, the Knoevenagel condensation was performed by the nucleophilic addition of active methylene of dimedone to the activated carbonyl group of aldehyde to produce intermediate I. On the other, intermediate II (enamine) was formed through the condensation of ammonia with catalyst-activated ethyl acetoacetate. Then, intermediate I performed a Michael addition with enamine to give intermediate III. Finally, the final product was synthesized by intramolecular cyclization of III followed by H_2_O elimination.

### 3.3. Efficiency of the catalyst

**Fig. 10:**
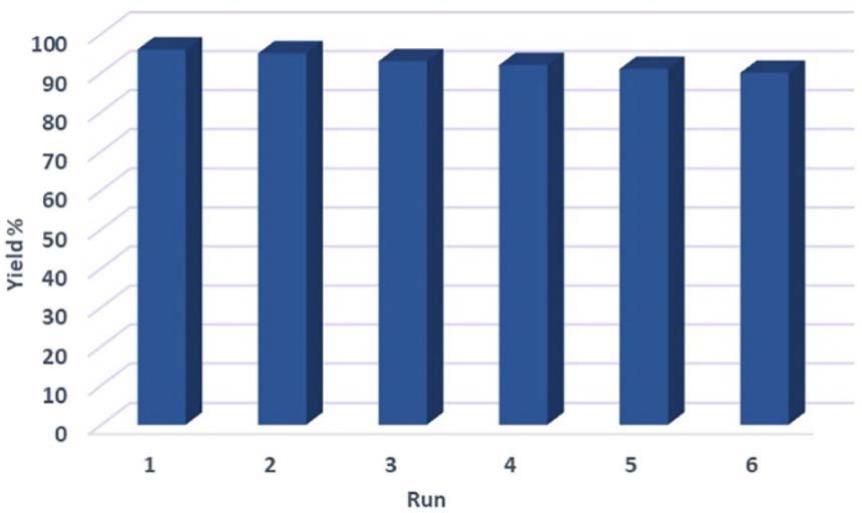
Reusability of Fe_3_O_4_@FSM-16-SO_3_/IL in the synthesis of 5a.

In order to investigate the heterogeneous or homogeneous nature of the Fe_3_O_4_@FSM-16-SO_3_/IL nanocatalyst, a leaching test was performed under optimum reaction conditions in the model reaction. To this end, the catalyst was separated by an external magnet after almost 50% of the reaction had taken place. Next, the residue of the reaction mixture under optimum conditions was stirred, but no significant progress in the reaction was observed. The result of this test confirms that the catalyst acts heterogeneously. To evaluate the recyclability and reusability of the Fe_3_O_4_@FSM-16-SO_3_/IL magnetic solid acid nanocatalyst, the reaction of benzaldehyde, dimedone, ethyl acetoacetate, ammonium acetate under optimum reaction conditions was carried. After completion of the reaction process, the catalyst was separated by the external magnetic field and was washed with ethanol, dried, and reused six times without any significant decrease in its catalytic activity (Fig. 10).

**Fig. 11:**
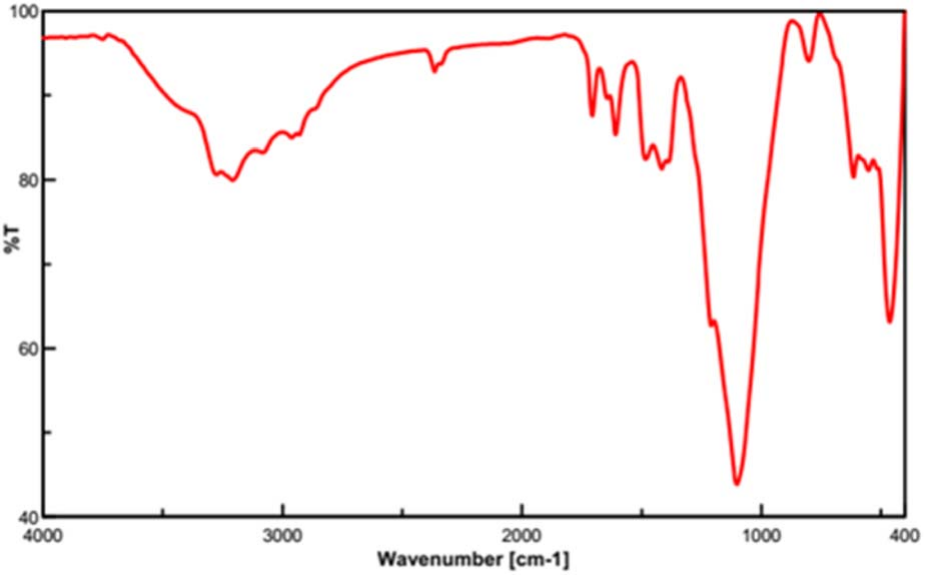
FT-IR spectrum of the recycled Fe_3_O_4_@FSM-16-SO_3_/IL catalyst.

**Fig. 12:**
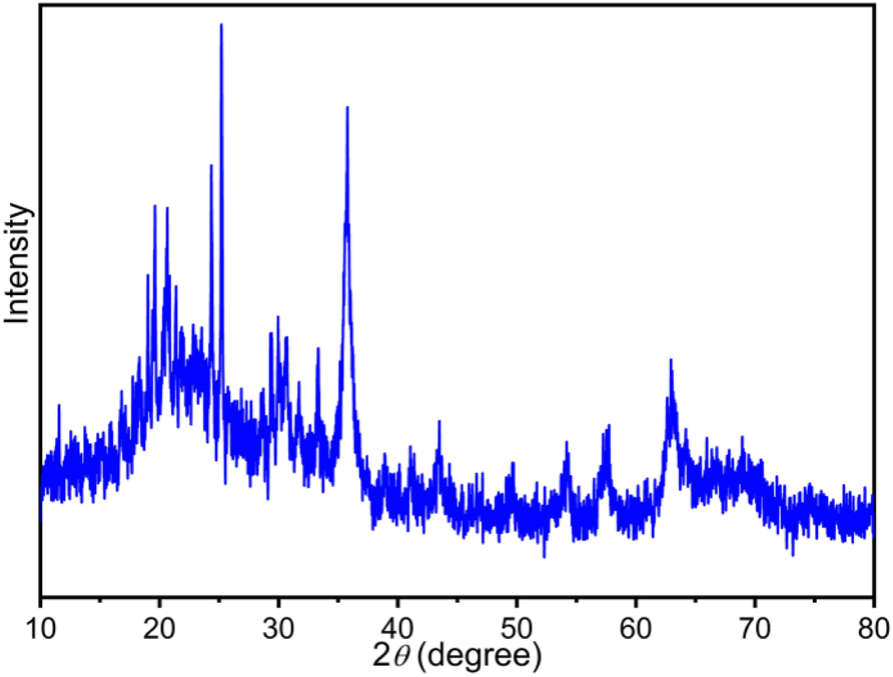
XRD pattern of the recycled Fe_3_O_4_@FSM-16-SO_3_/IL catalyst.

In the following, the FT-IR spectrum of the recovered Fe_3_O_4_@FSM-16-SO_3_/IL nanocatalyst is shown in Fig. 11. This analysis confirms the high strength and stability of the catalyst structure after recycling. Also, the XRD diffraction pattern of the recovered catalyst was analyzed. As shown, the relative intensity and position of all peaks, and the structural stability were confirmed (Fig. 12).

Next, the comparison of the performance of the Fe_3_O_4_@FSM-16-SO_3_/IL catalyst was investigated with other reported catalysts for the synthesis of polyhydroquinoline derivatives was investigated. Based on the results in Table 3, the Fe_3_O_4_@FSM-16-SO_3_/IL nanocatalyst is comparable with other catalysts in terms of recycling times and reusability, short reaction time, amount of the catalyst, reaction temperature, type of solvent, and product yield.

**Table 3:** Comparison results of Fe_3_O_4_@FSM-16-SO_3_/IL with other catalysts for the synthesis of polyhydroquinoline derivatives

Catalyst	Conditions	Time (min)	Yield^a^ (%)
Fe_3_O_4_@SiO_2_/ZnCl_2_	Cat. (0.05), solvent-free, 110 °C	25	94 (ref. 61)
SPPN	Cat. (6 mg), EtOH, MW	8	96 (ref. 62)
TrzMOP	Cat. (8 mg), EtOH, 120 °C, MW	10	98 (ref. 63)
[TBA]_2_[W_6_O_19_]	Cat. (0.07 mmol), solvent-free, 110 °C	20	93 (ref. 64)
GSA@MNPs	Cat. (0.05 mg), EtOH, reflux	240	90 (ref. 65)
H_2_SO_4_	Cat. (0.004 g), EtOH, reflux	720	—b
Fe_3_O_4_@FSM-16-SO_3_/IL	Cat. (0.004 g), EtOH, reflux	5	98^b^

a Isolated yields.

b This work.

## 4. Conclusions

This study presents a new ionic liquid based on magnetic FSM-16 nanocomposite and its catalytic application in the synthesis of polyhydroquinoline derivatives. The FE-SEM and TEM images of the Fe_3_O_4_@FSM-16-SO_3_/IL catalyst showed a spherical morphology with a highly ordered structure. Furthermore, the XRD, TGA, EDX, and FT-IR analyses confirmed the high stability, and also the immobilization of the ionic liquid on the magnetic mesoporous framework. Also, the VSM analysis showed well the magnetic properties of this nanocomposite. Some of the remarkable advantages of this catalyst are short reaction time, excellent product yield, and chemical stability. More importantly, the catalyst can be recovered using an external magnet and reused 6 times without significant loss of catalytic activity.

## Conflicts of interest

There are no conflicts to declare.

## Supplementary Material

RA-013-D3RA04953A-s001
