# Supplementary material for: Ionic liquid immobilized on modified magnetic FSM-16: an efficient and magnetically recoverable nanocatalyst
Source: RSC Adv. 2023 Oct 25;13(44):31252–62. doi: 10.1039/d3ra04953a (PMC10599354; doi:10.1039/d3ra04953a)
Supplement: RA-013-D3RA04953A-s001 [file RA-013-D3RA04953A-s001.pdf]

Electronic Supplementary Material (ESI) for RSC Advances.
This journal is © The Royal Society of Chemistry 2023

Supporting Information

Ionic liquid immobilized on modified magnetic FSM-16: An efficient and magnetically recoverable nanocatalyst

Azar Jahanbakhshi and Mahnaz Farahi*

Department of Chemistry, Yasouj University, P. O. Box 353, Yasouj 75918-74831, Iran

**Corresponding author: E-mail: farahimb@yu.ac.ir*

**Spectral Data FT-IR**	
**5a**	3288 (N-H, stretching), 3027 (C-H, Ar), 2962 (C-H stretching), 1692 (C=O, ester), 1606(C=O, ketone), 1492(C=C, Ar), 1249 (C-N), 1160 (C-O, ester), 979 cm^{-1}.
**5b**	3274 (N-H, stretching), 3066 (C-H, Ar), 2962 (C-H stretching), 1704 (C=O, ester), 1604 (C=O, ketone), 1496 (C=C, Ar), 1249(C-N), 1172(C-O, ester), 883 cm^{-1}.
**5c**	3274 (N-H, stretching), 3070 (C-H, Ar), 2958 (C-H stretching), 1708 (C=O, ester), 1639 (C=O, ketone), 1492 (C=C, Ar), 1218 (C-N), 1092(C-O, ester), 833 cm^{-1}.
**5d**	3282 (N-H, stretching), 3073(C-H, Ar), 2958 (C-H stretching), 1681(C=O, ester), 1604(C=O, ketone), 1488 (C=C, Ar), 1145 (C-N), 1072 (C-O, ester), 883 cm^{-1}.
**5e**	3291(N-H, stretching), 3074(C-H, Ar), 2968(C-H stretching), 1707(C=O, ester), 1659(C=O, ketone), 1486(C=C, Ar), 1213(C-N), 1041 (C-O, ester), 797 $cm^{-1.}$
**5f**	3289 (N-H, stretching), 3068(C-H, Ar), 2962(C-H stretching), 1702(C=O, ester), 1650(C=O, ketone), 1494(C=C, Ar), 1220(C-N), 1089 (C-O, ester), 763 cm^{-1}.
**5g**	3237 (N-H, stretching), 3053(C-H, Ar), 2936(C-H stretching), 1702(C=O, ester), 1643(C=O, ketone), 1489(C=C, Ar), 1221(C-N), 1089 (C-O, ester), 783 cm^{-1}.
**5h**	3226 (N-H, stretching), 3077(C-H, Ar), 2976(C-H stretching), 1700(C=O, ester), 1648(C=O, ketone), 1494(C=C, Ar), 1230(C-N), 1075 (C-O, ester) cm^{-1}.
**5i**	3440 (N-H, stretching), 3087(C-H, Ar), 2958(C-H stretching), 1717(C=O, ester), 1634(C=O, ketone), 1498(C=C, Ar), 1243(C-N), 1222(C-O), 1065 (C-O, ester) cm^{-1}.

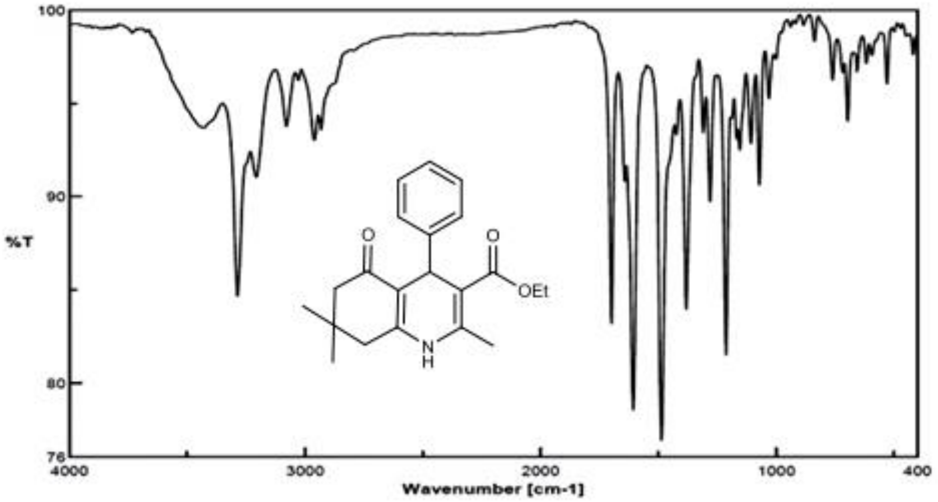

FT-IR spectrum of Ethyl-2,7,7-trimethyl-5-oxo-4-phenyl-1,4,5,6,7,8-hexahydroquinoline-3-carboxylate (**5a**).

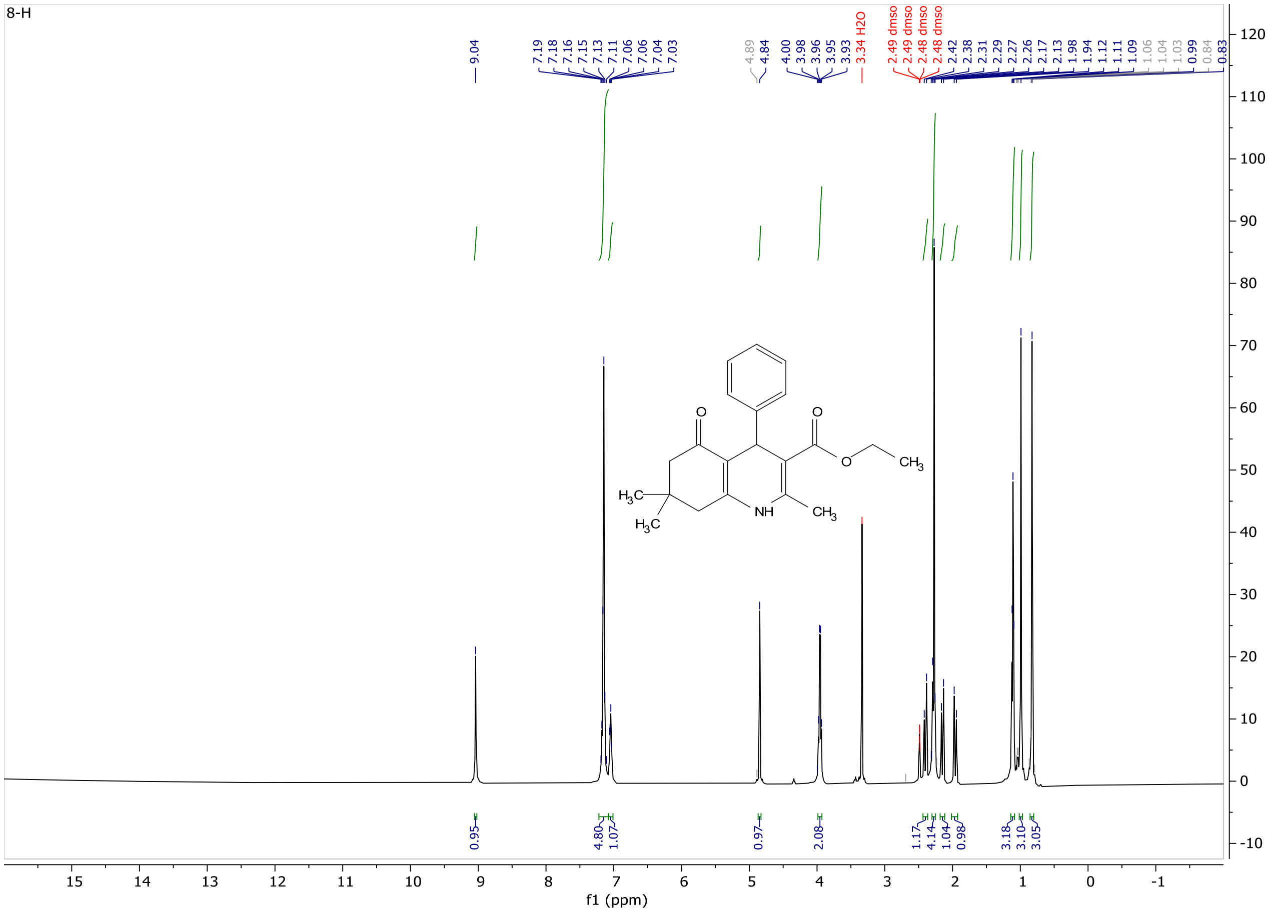

^{1}H NMR spectrum of Ethyl-2,7,7-trimethyl-5-oxo-4-phenyl-1,4,5,6,7,8-hexahydroquinoline-3-carboxylate (**5a**)

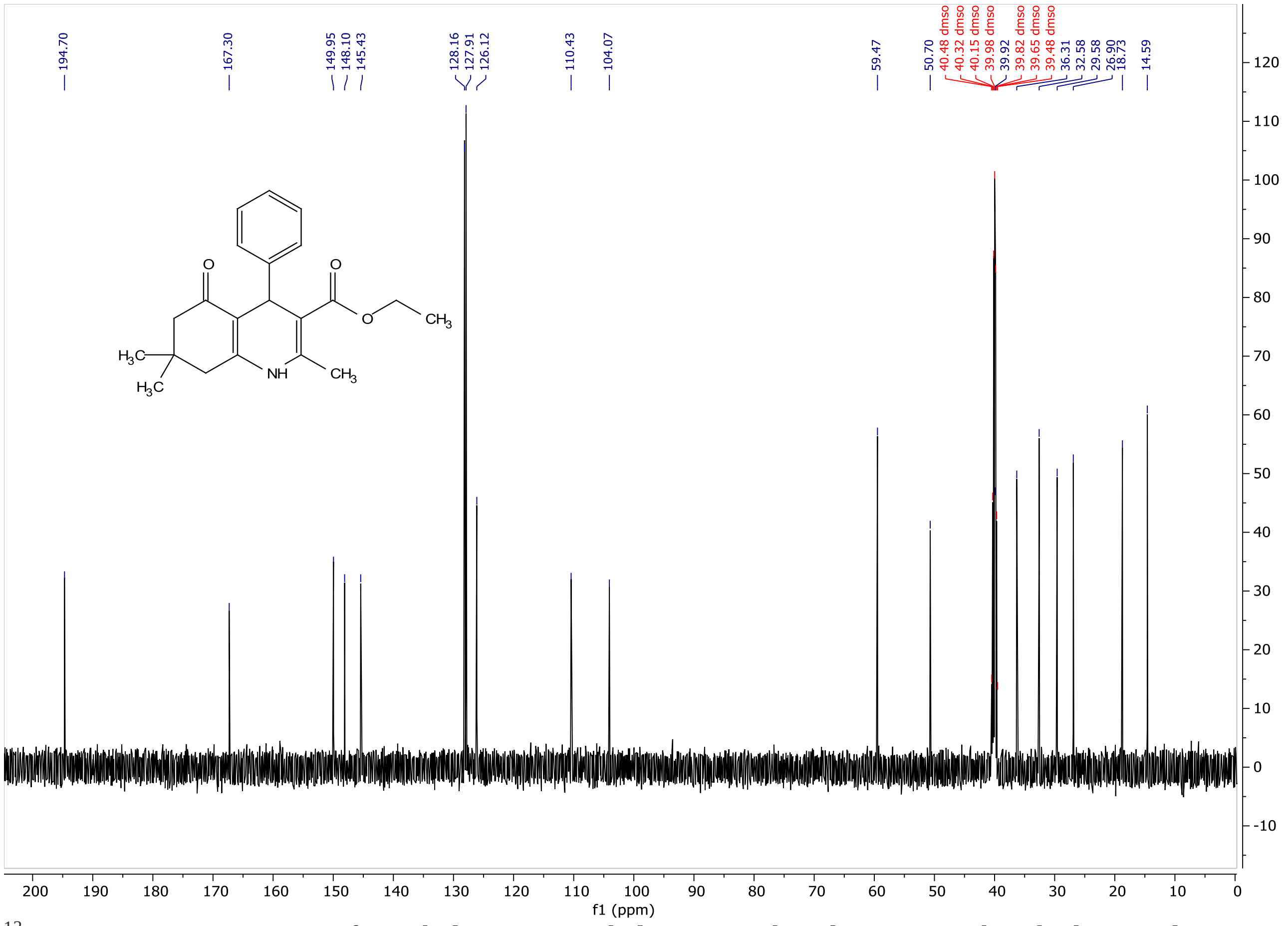

^{13}C NMR spectrum of Ethyl-2,7,7-trimethyl-5-oxo-4-phenyl-1,4,5,6,7,8-hexahydroquinoline-3-carboxylate (**5a**)

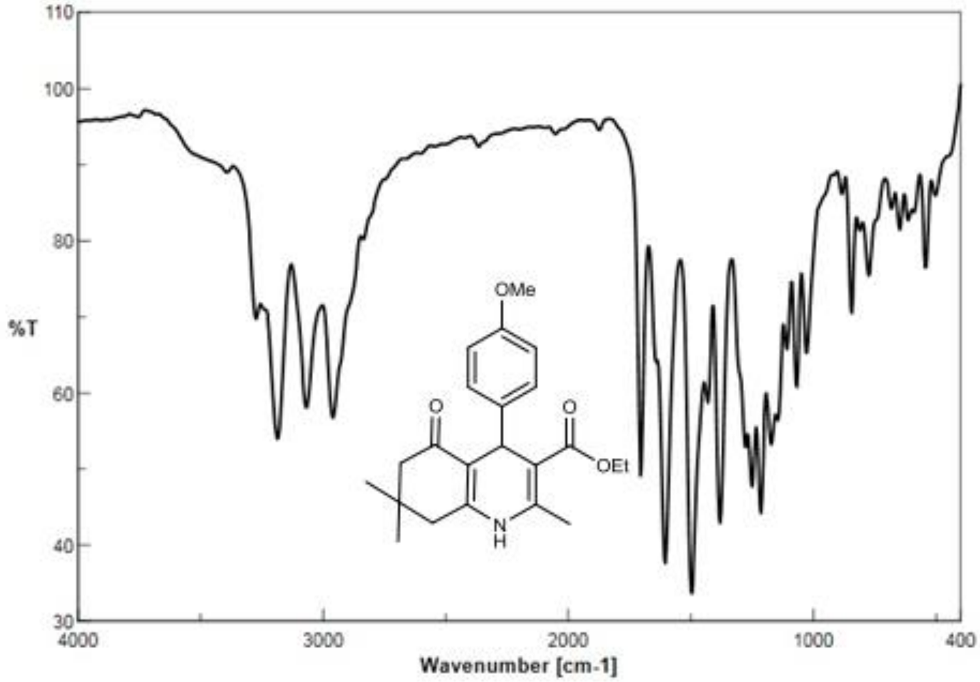

FT-IR spectrum of Ethyl-4-(4-methoxyphenyl)-2,7,7-trimethyl-5-oxo-1,4,5,6,7,8-hexahydro quinoline-3-carboxylate (**5b**).

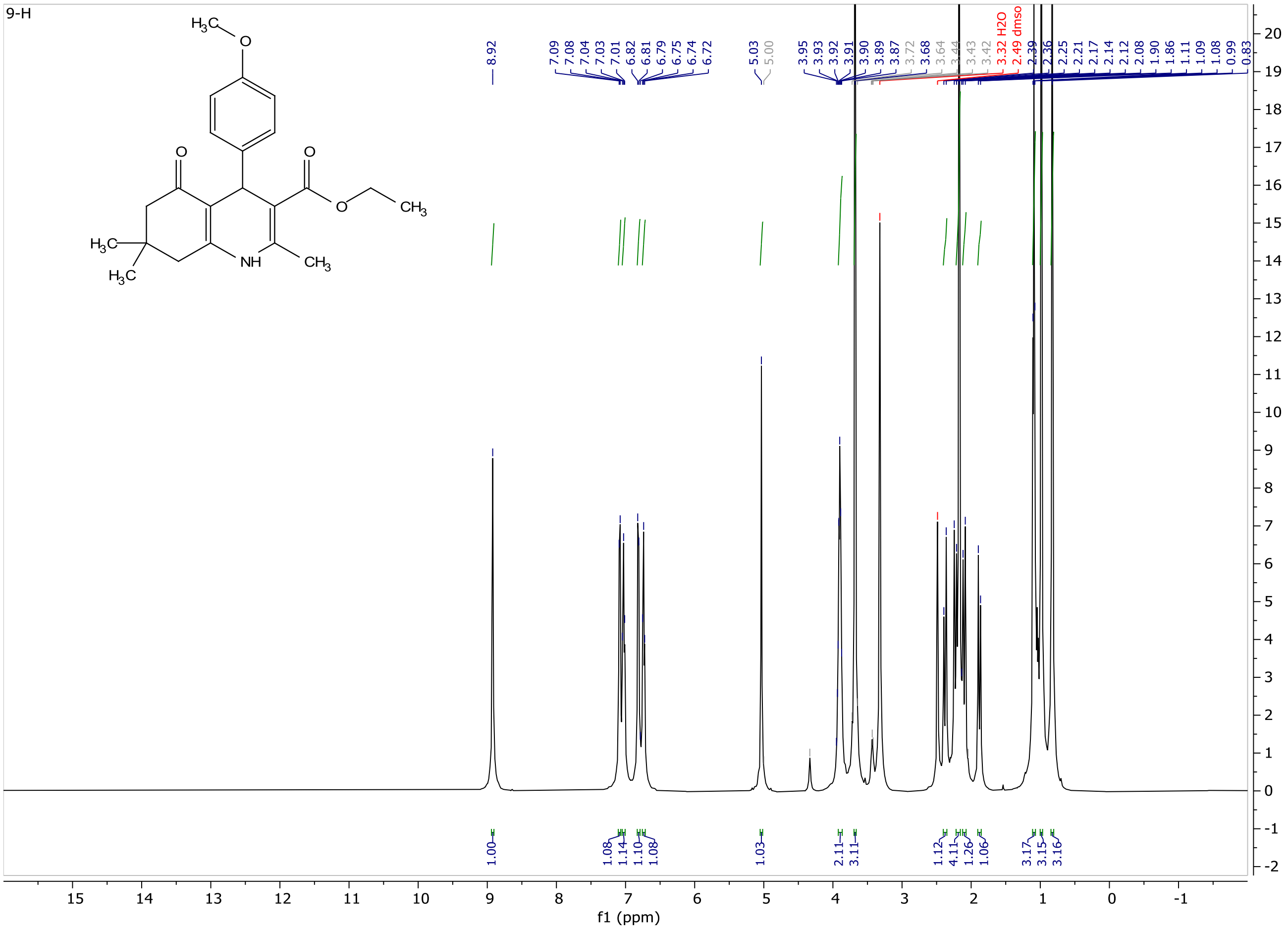

^{1}H NMR spectrum of Ethyl-4-(4-methoxyphenyl)-2,7,7-trimethyl-5-oxo-1,4,5,6,7,8-hexahydro quinoline-3-carboxylate (**5b**).

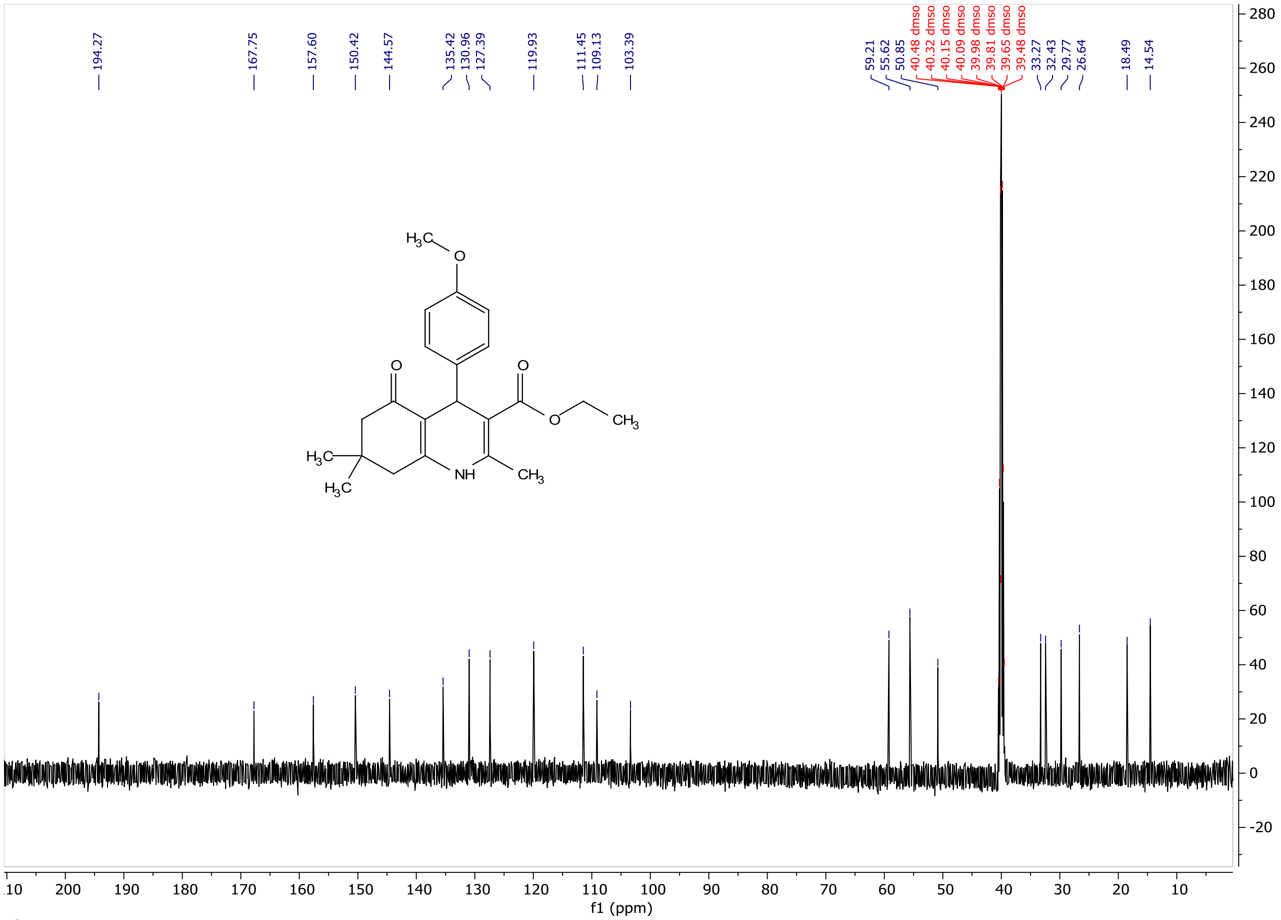

^{13}C NMR spectrum of Ethyl-4-(4-methoxyphenyl)-2,7,7-trimethyl-5-oxo-1,4,5,6,7,8-hexahydro quinoline-3-carboxylate (**5b**).

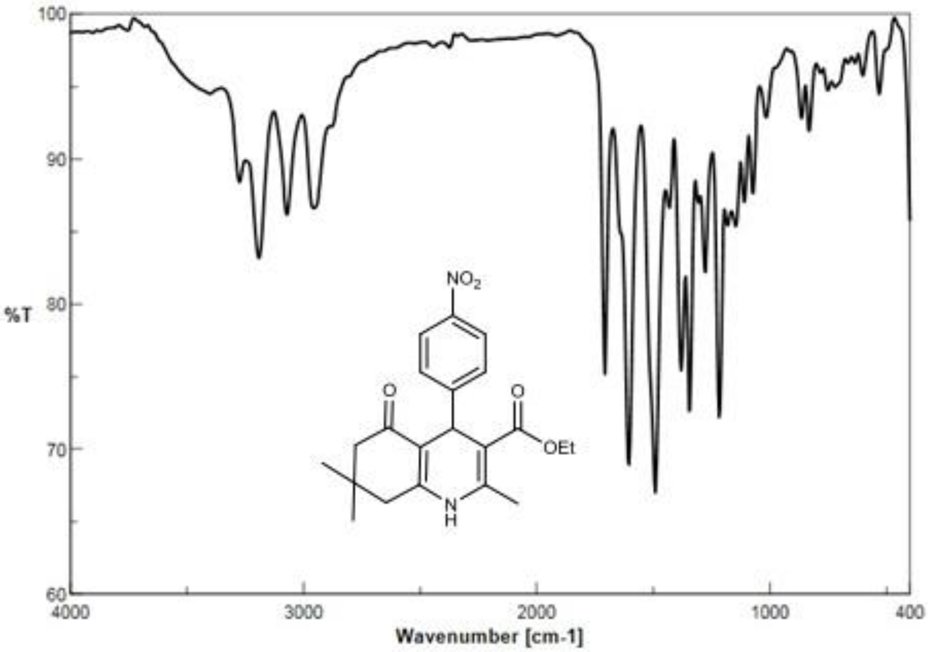

FT-IR spectrum of Ethyl2,7,7-trimethyl-4-(4-nitrophenyl)-5-oxo-1,4,5,6,7,8-hexahydro quinoline-3-carboxylate (**5c**).

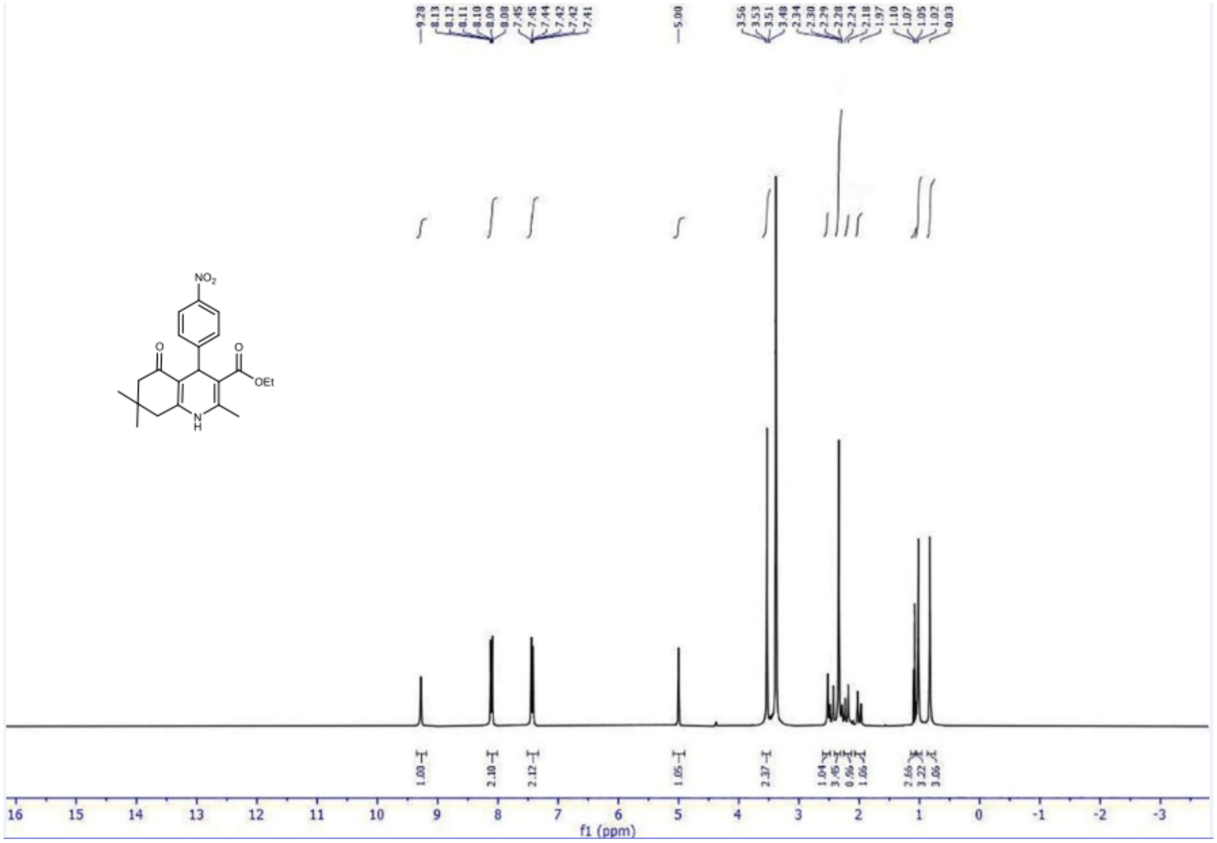

^{1}H NMR spectrum of Ethyl2,7,7-trimethyl-4-(4-nitrophenyl)-5-oxo-1,4,5,6,7,8-hexahydro quinoline-3-carboxylate (**5c**).

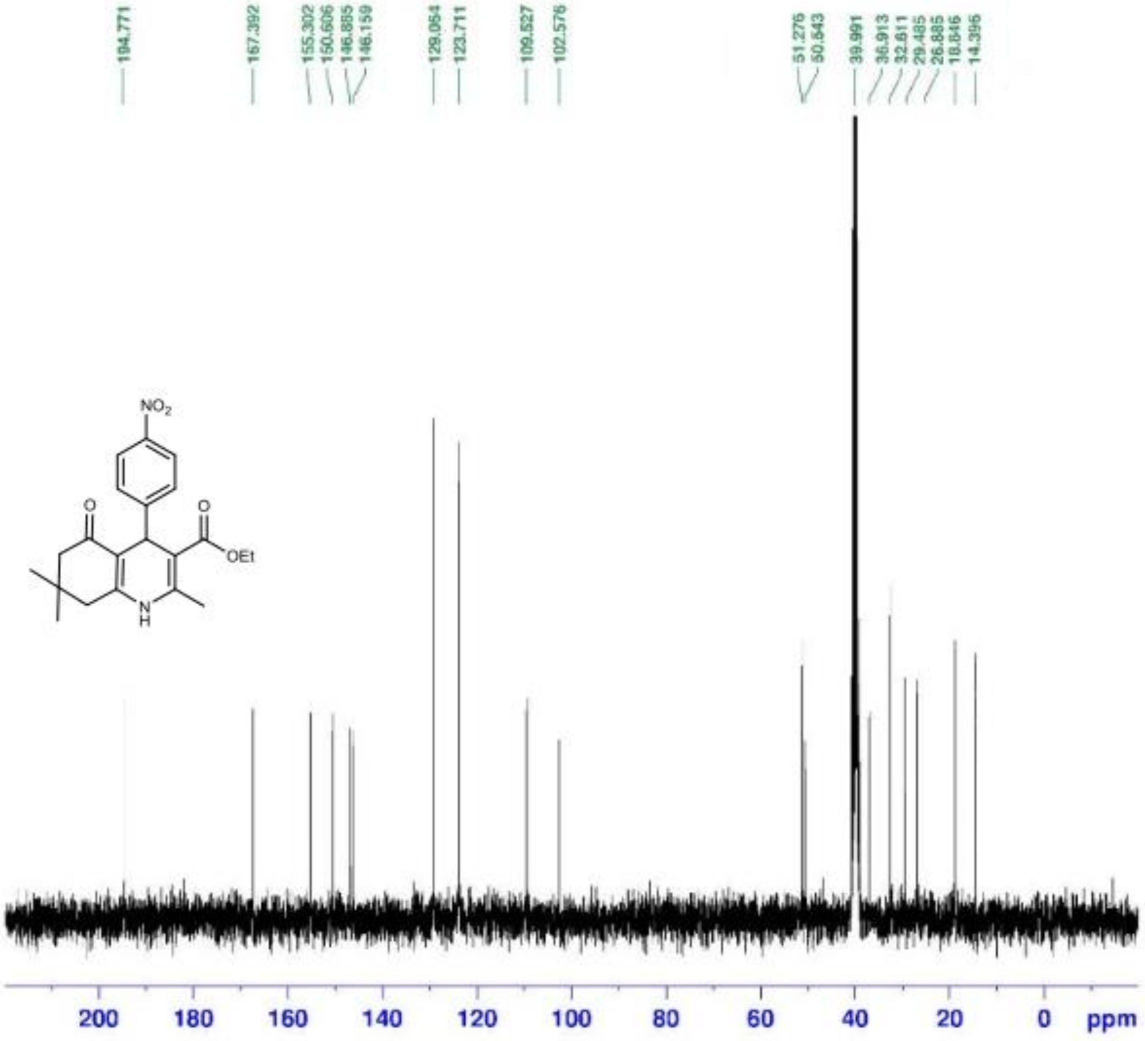

^{13}C NMR spectrum of Ethyl2,7,7-trimethyl-4-(4-nitrophenyl)-5-oxo-1,4,5,6,7,8-hexahydro quinoline-3-carboxylate (**5c**).

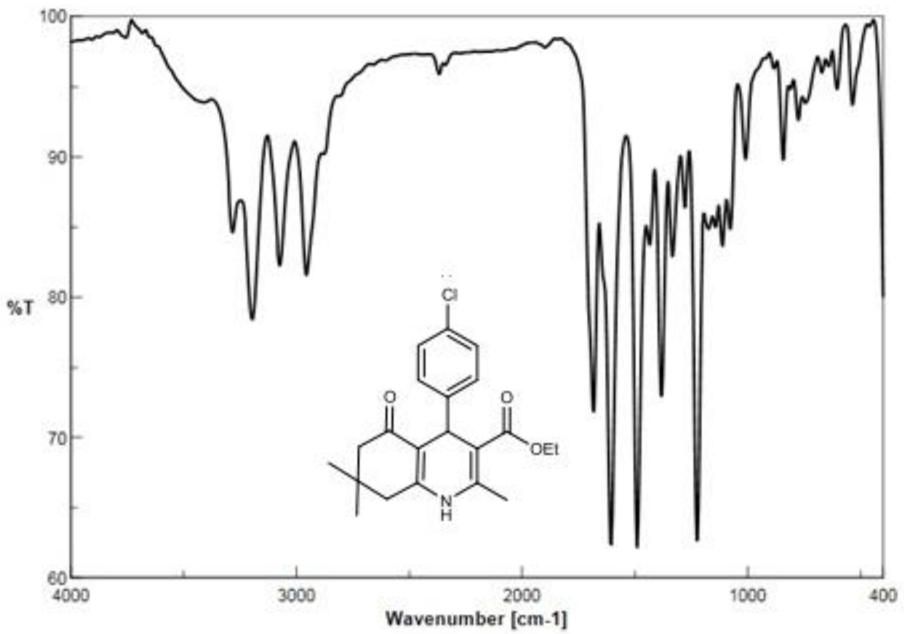

FT-IR spectrum of Eethyl-4-(4-chlorophenyl)-2,7,7-trimethyl-5-oxo-1,4,5,6,7,8-hexahydro quinoline-3-carboxylate (**5d**).

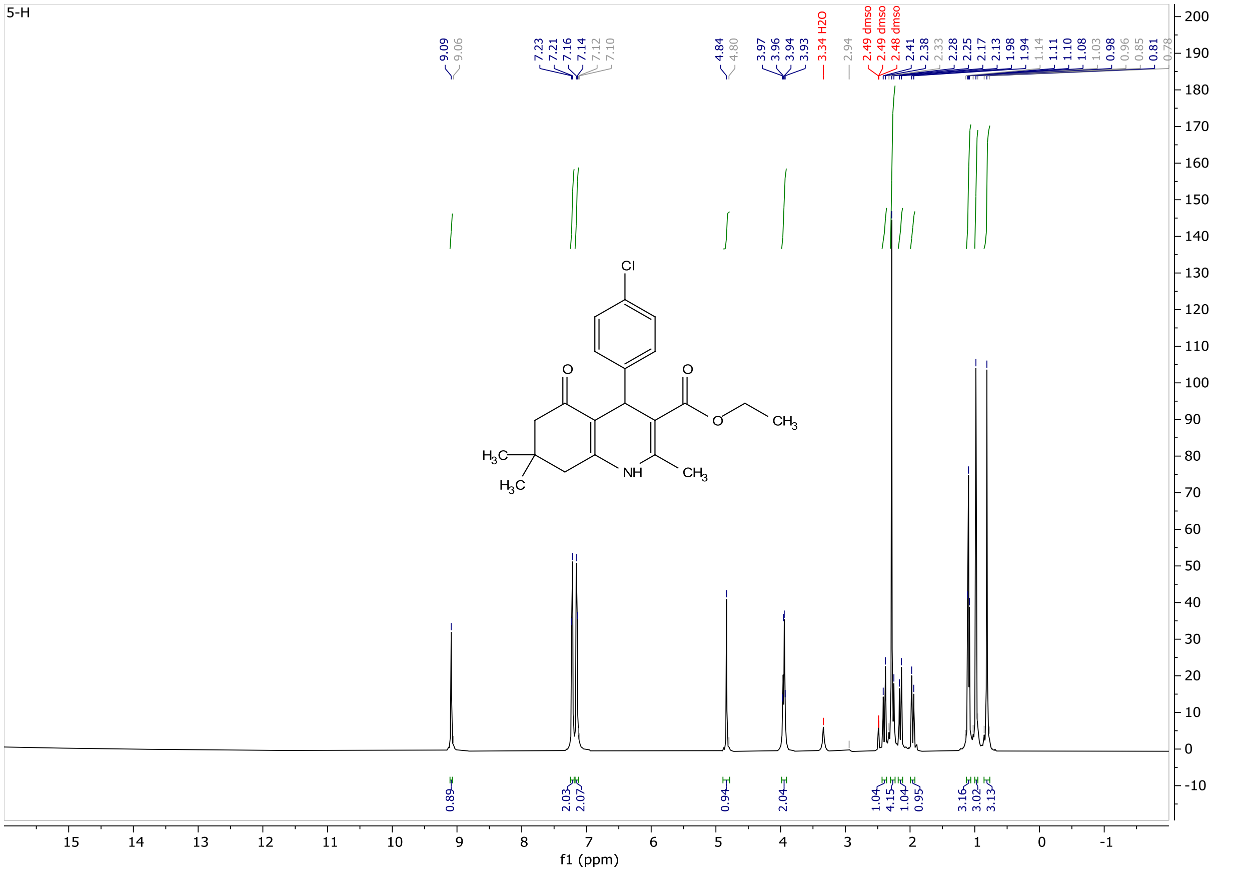

^{1}H NMR spectrum of Eethyl-4-(4-chlorophenyl)-2,7,7-trimethyl-5-oxo-1,4,5,6,7,8-hexahydro quinoline-3-carboxylate(**5d**).

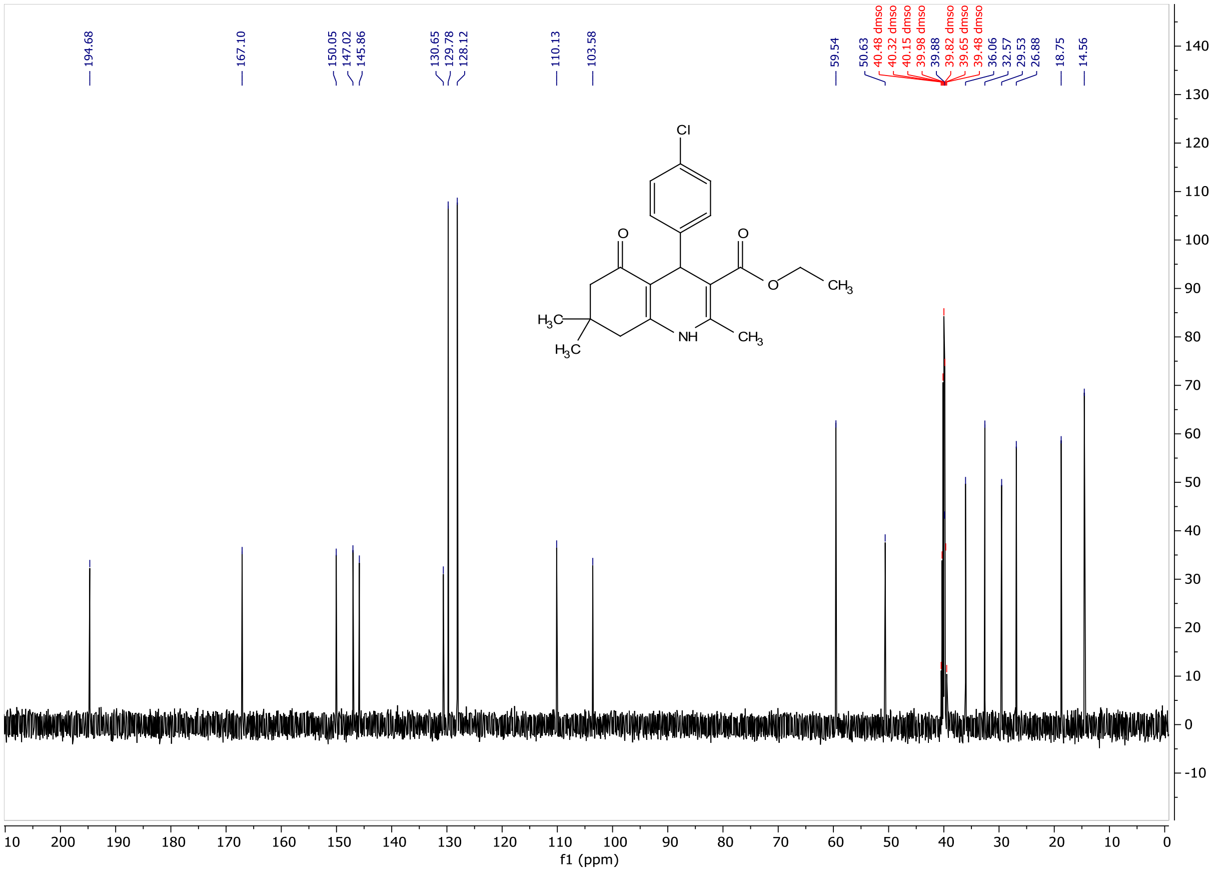

^{13}C NMR spectrum of Eethyl-4-(4-chlorophenyl)-2,7,7-trimethyl-5-oxo-1,4,5,6,7,8-hexahydro quinoline-3-carboxylate (**5d**).

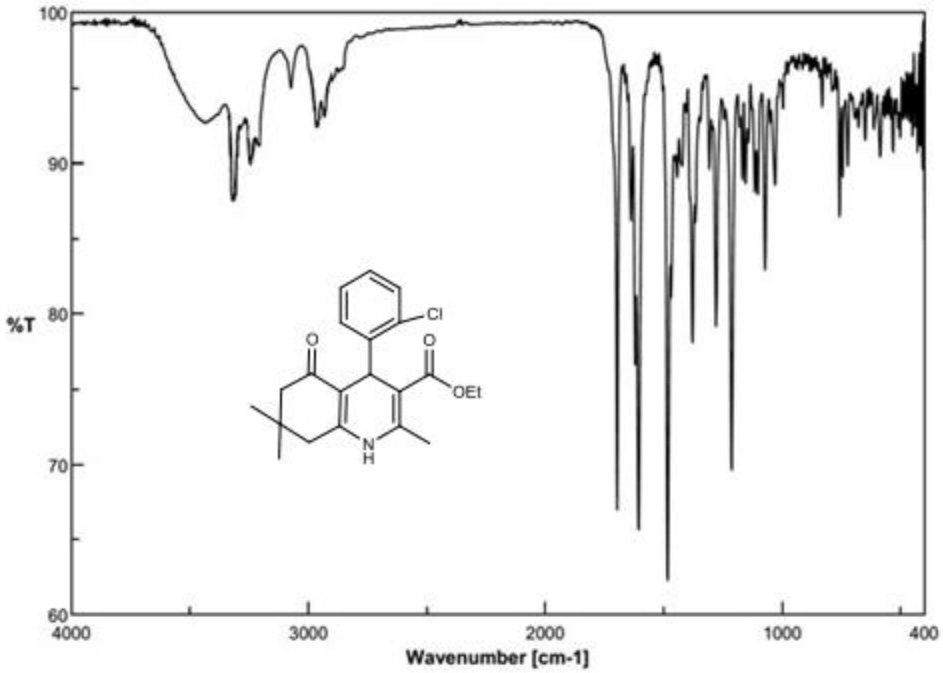

FT-IR spectrum of Eethyl-4-(2-chlorophenyl)-2,7,7-trimethyl-5-oxo-1,4,5,6,7,8-hexahydro quinoline-3-carboxylate (**5e**).

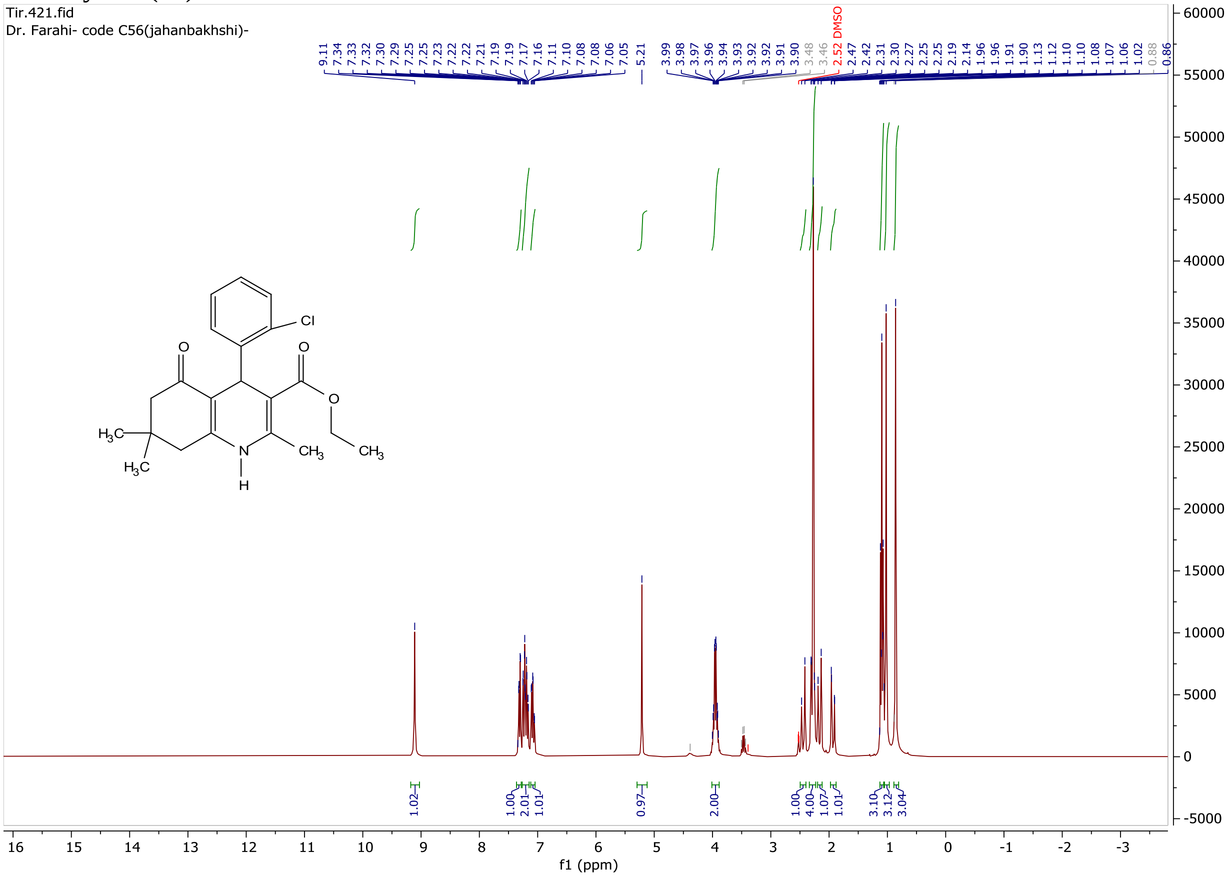

^{1}H NMR spectrum of Eethyl-4-(2-chlorophenyl)-2,7,7-trimethyl-5-oxo-1,4,5,6,7,8-hexahydro quinoline-3-carboxylate (**5e**).

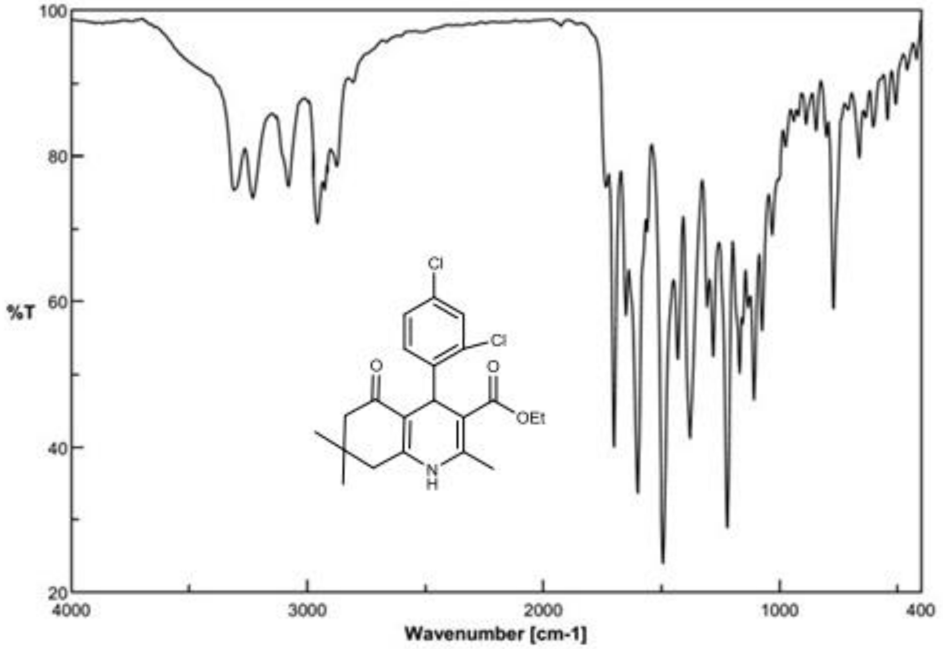

FT-IR spectrum of Ethyl-4-(2,4-dichlorophenyl)-2,7,7-trimethyl-5-oxo-1,4,5,6,7,8-hexahydro quinoline-3-carboxylate (**5f**).

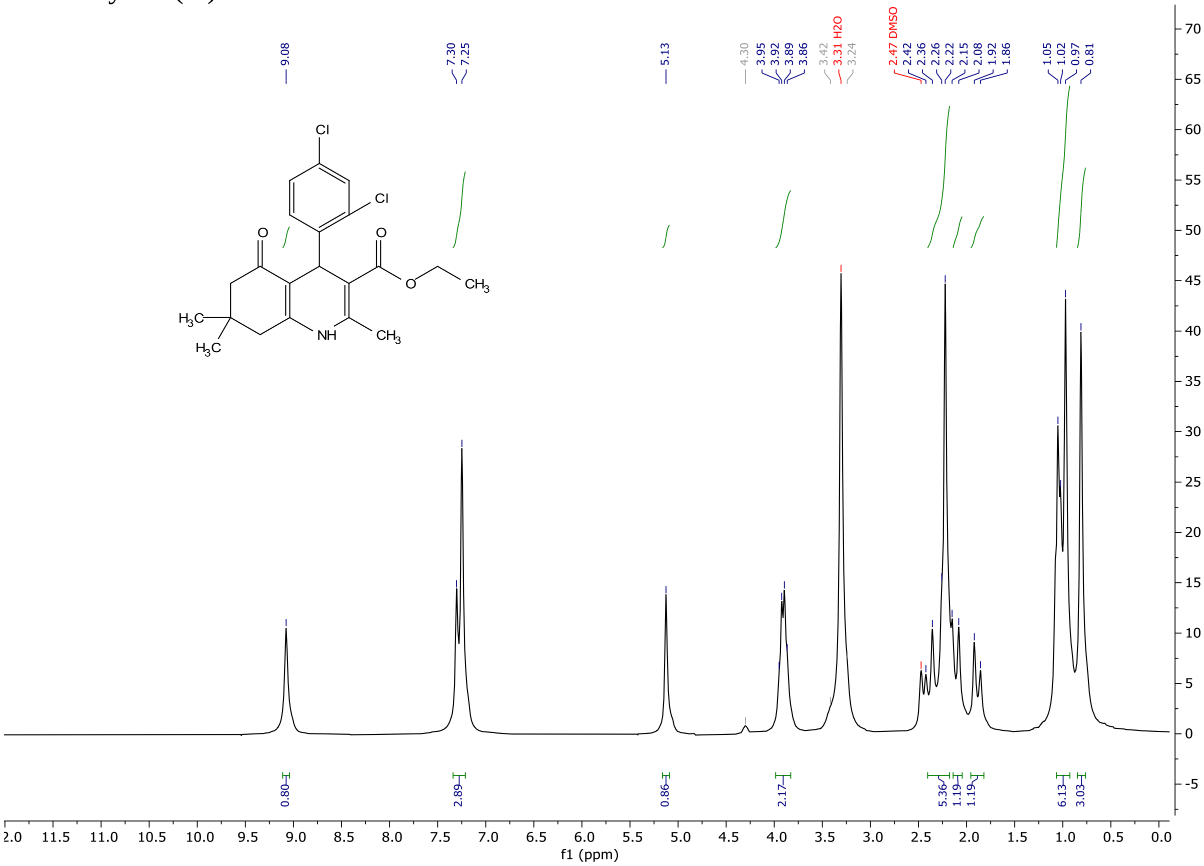

^{1}H NMR spectrum of Ethyl-4-(2,4-dichlorophenyl)-2,7,7-trimethyl-5-oxo-1,4,5,6,7,8-hexahydro quinoline-3-carboxylate (**5f**).

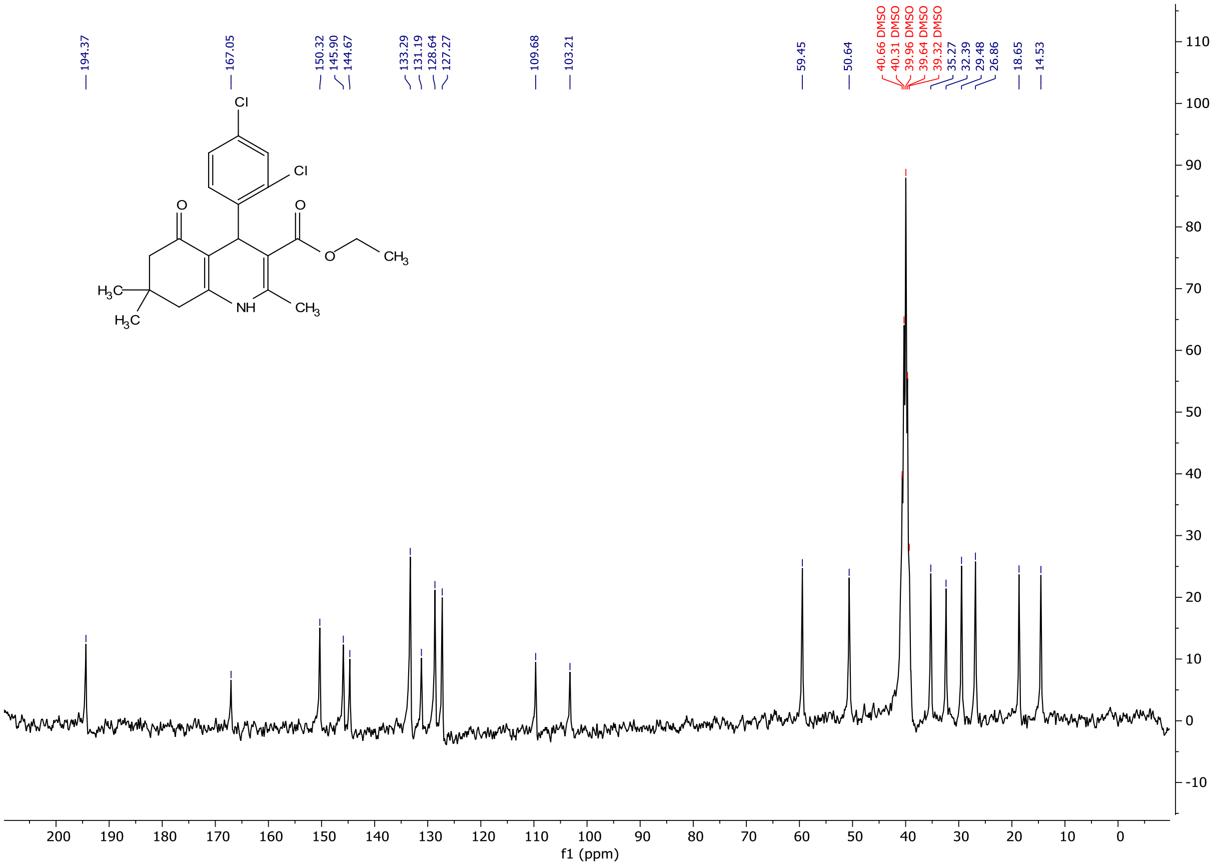

^{13}C NMR spectrum of Ethyl-4-(2,4-dichlorophenyl)-2,7,7-trimethyl-5-oxo-1,4,5,6,7,8-hexahydro quinoline-3-carboxylate (**5f**).

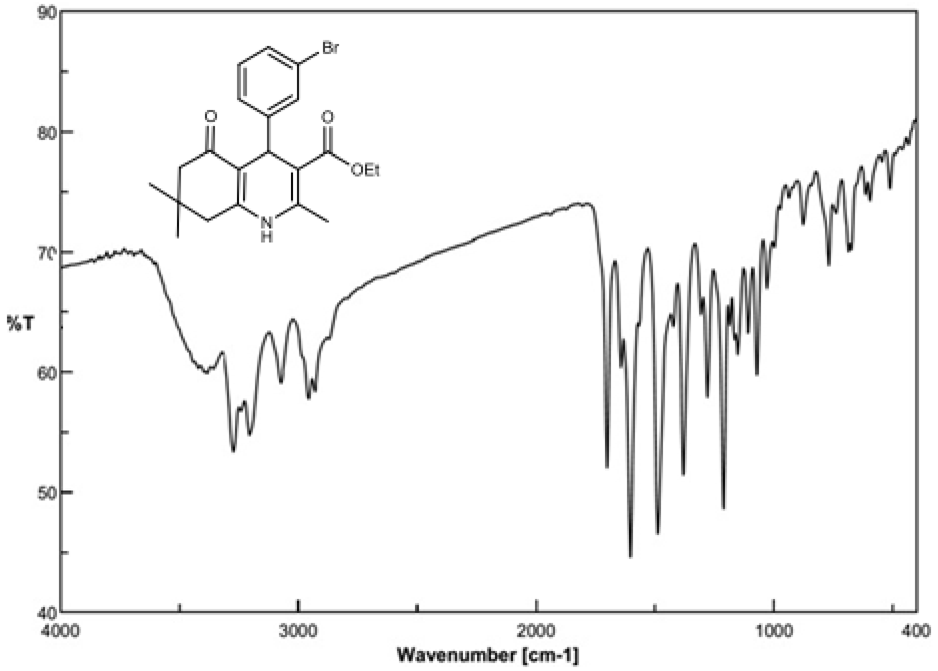

FT-IR spectrum of Ethyl-4-(3-bromophenyl)-2,7,7-trimethyl-5-oxo-1,4,5,6,7,8-hexahydro quinoline-3-carboxylate (**5g**).

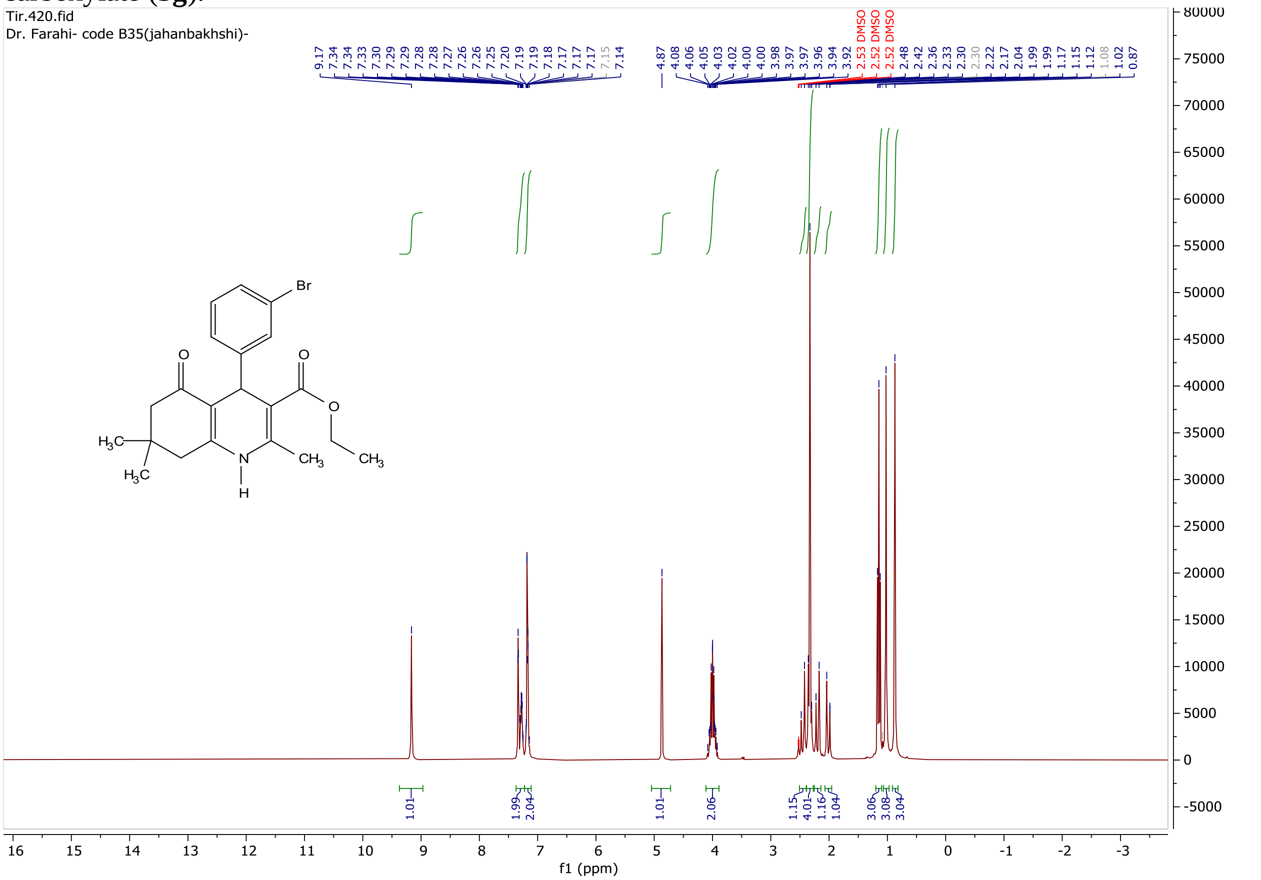

^{1}H NMR spectrum of Ethyl-4-(3-bromophenyl)-2,7,7-trimethyl-5-oxo-1,4,5,6,7,8-hexahydro quinoline-3-carboxylate (**5g**).

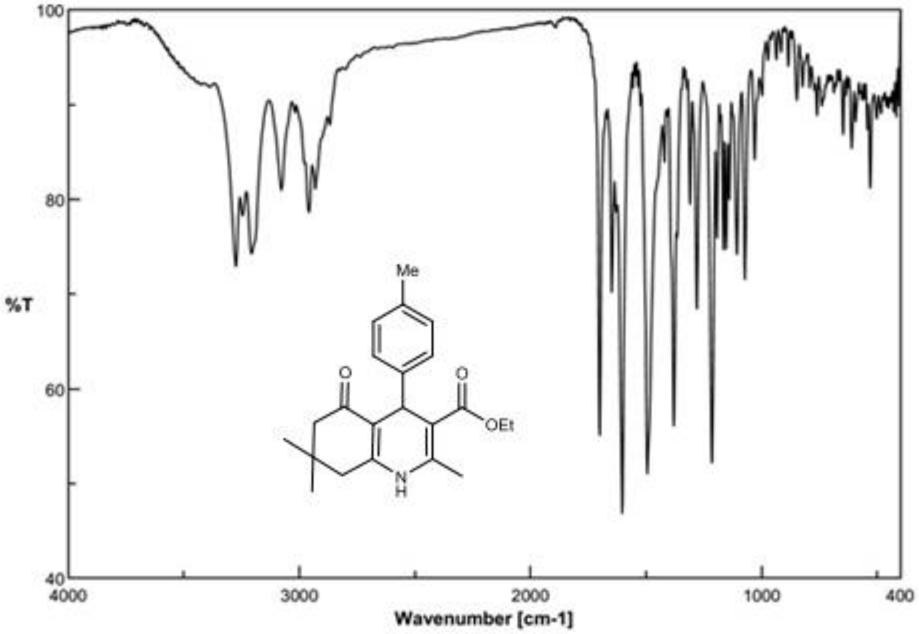

FT-IR spectrum of Ethyl-2,7,7-trimethyl-5-oxo-4-(p-tolyl)-1,4,5,6,7,8-hexahydroquinoline-3-carboxylate (**5h**).

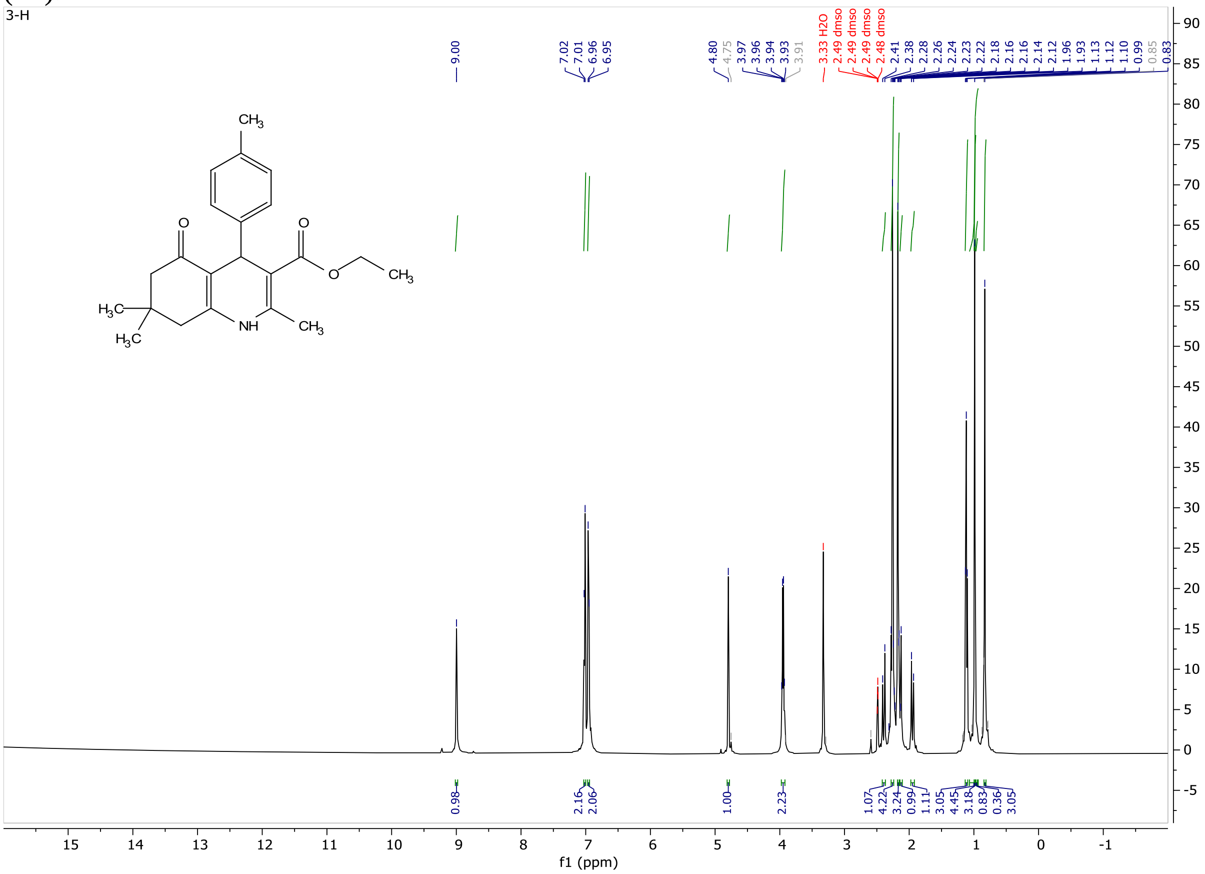

^{1}H NMR spectrum of Ethyl-2,7,7-trimethyl-5-oxo-4-(p-tolyl)-1,4,5,6,7,8-hexahydroquinoline-3-carboxylate (**5h**).

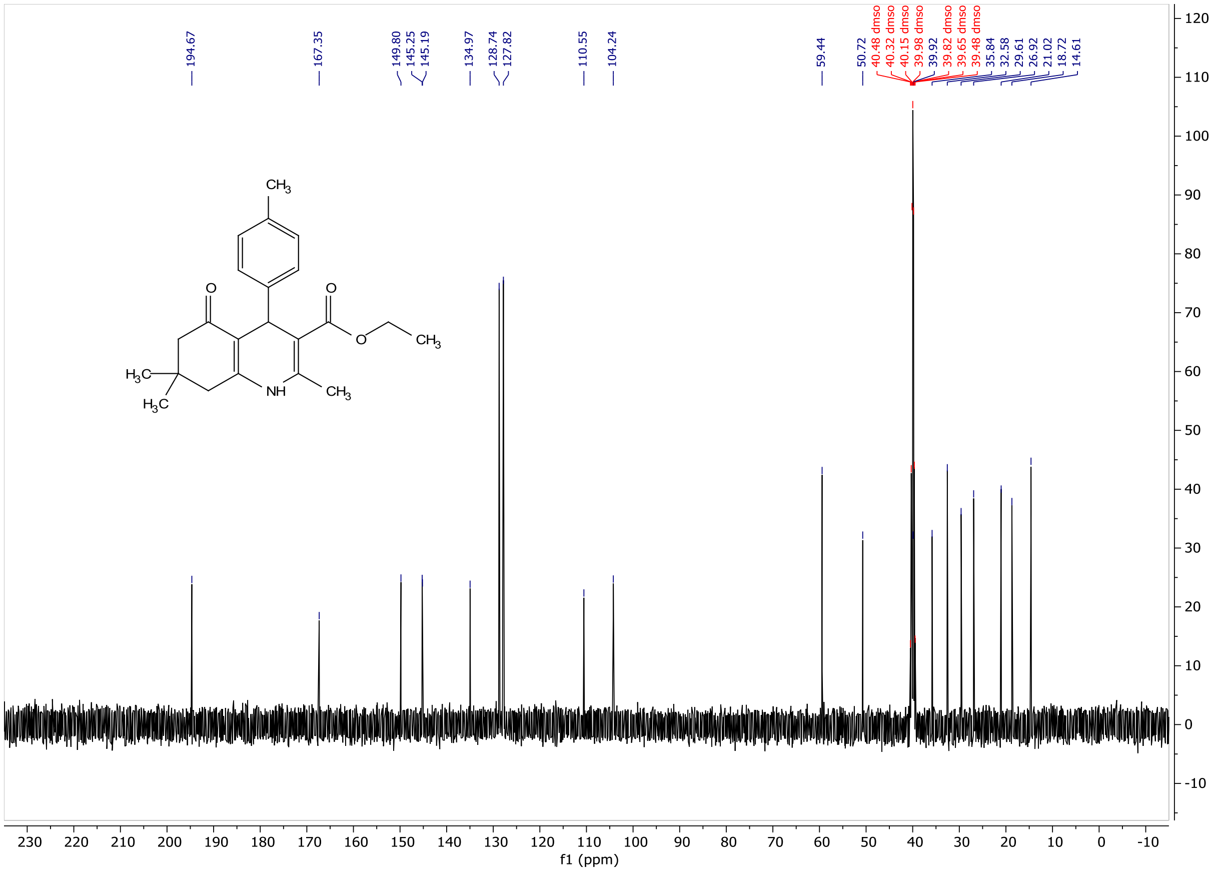

^{13}C NMR spectrum of Ethyl-2,7,7-trimethyl-5-oxo-4-(p-tolyl)-1,4,5,6,7,8-hexahydroquinoline-3-carboxylate (**5h**).

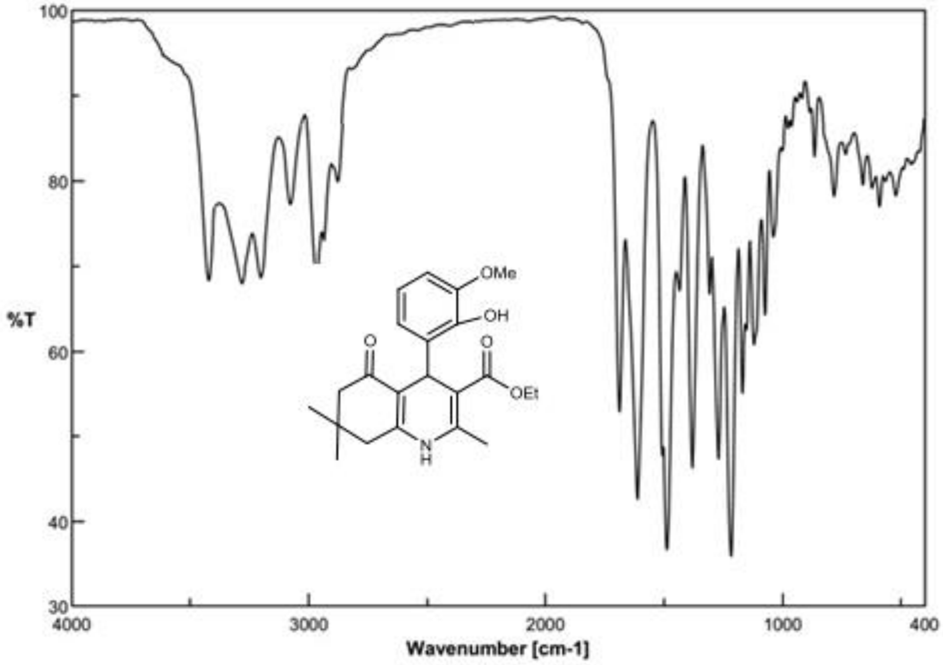

FT-IR spectrum of Ethyl-4-(2-hydroxy-3-methoxyphenyl)-2,7,7-trimethyl-5-oxo-1,4,5,6,7,8-hexa hydroquinoline-3-carboxylate (**5i**).

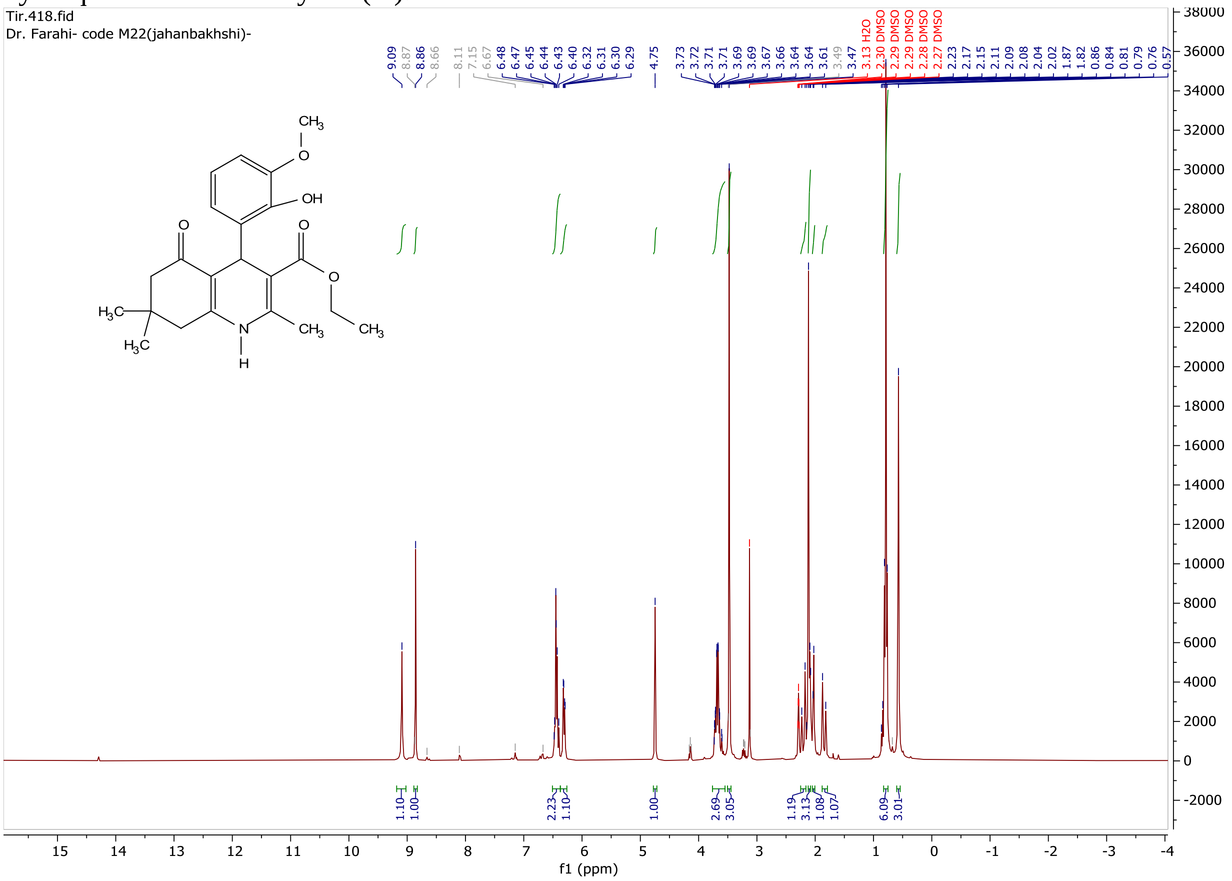

^{1}H NMR spectrum of Ethyl-4-(2-hydroxy-3-methoxyphenyl)-2,7,7-trimethyl-5-oxo-1,4,5,6,7,8-hexa hydroquinoline-3-carboxylate (**5i**).
